# Bubble-Manipulated Local Drug Release from a Smart Thermosensitive Cerasome for Dual-Mode Imaging Guided Tumor Chemo-Photothermal Therapy

**DOI:** 10.7150/thno.36762

**Published:** 2019-10-18

**Authors:** Suhui Sun, Sujuan Sun, Yan Sun, Ping Wang, Jianlun Zhang, Wenjing Du, Shumin Wang, Xiaolong Liang

**Affiliations:** 1 Department of Ultrasound, Peking University Third Hospital, Beijing, China; 2 Ordos Center Hospital, Ordos 017000, Inner Mongolia, China

**Keywords:** controlled drug release, combined therapy, cerasome, liposome, bubble responsive

## Abstract

Thermosensitive liposomes have demonstrated great potential for tumor-specific chemotherapy. Near infrared (NIR) dyes loaded liposomes have also shown improved photothermal effect in cancer theranostics. However, the instability of liposomes often causes premature release of drugs or dyes, impeding their antitumor efficacy. Herein, we fabricated a highly stable thermo-responsive bubble-generating liposomal nanohybrid cerasome with a silicate framework, combined with a NIR dye to achieve NIR light stimulated, tumor-specific, chemo-photothermal synergistic therapy.

**Methods:** In this system, NIR dye of 1,1'-Dioctadecyl-3,3,3',3'- Tetramethylindotricarbocyanine iodide (DiR) with long carbon chains was self-assembled with a cerasome-forming lipid (CFL) to encapsulate ammonium bicarbonate (ABC), which was further used for actively loading doxorubicin (DOX), affording a thermosensitive and photosensitive DOX-DiR@cerasome (ABC).

**Results:** The resulting cerasome could disperse well in different media. Upon NIR light mediated thermal effect, ABC was decomposed to generate CO_2_ bubbles, resulting in a permeable channel in the cerasome bilayer that significantly enhanced DOX release. After intravenous injection into tumor-bearing mice, DOX-DiR@cerasome (ABC) could be efficiently accumulated at the tumor tissue, as monitored by DiR fluorescence, lasting for more than 5 days. NIR light irradiation was then performed at 36h to locally heat the tumors, resulting in immediate CO_2_ bubble generation, which could be clearly detected by ultrasound imaging, facilitating the monitoring process of controlled release of the drug. Significant antitumor efficacy could be obtained for the DOX-DiR@cerasome (ABC) + laser group, which was further confirmed by tumor tissue histological analysis.

## 1. Introduction

Liposomes have a nanostructure resembling a cell membrane with both a lipid bilayer and an aqueous core and therefore, hydrophobic and hydrophilic drugs can be efficiently loaded in the lipid bilayer and the aqueous core, respectively 1. Liposomal drugs with improved pharmacokinetics and biodistribution often show good biocompatibility and improved drug delivery to the target tissue 2-4, and thus have received widespread attention since their development as drug carriers in 1970 5. Since then, many liposomal drugs have been developed and are commercially available after being approved by the FDA for cancer treatment, such as Abraxane, Mariqbo, Doxil and DaunoXome 6, 7. However, poor bioavailability and limited drug efficacy still limits their clinical application 8, 9. Therefore, liposomes with an actively triggered drug release function have been desired to improve their therapeutic effect.

A thermo-responsive liposomal formulation (ThermoDox), consisting of three lipid components—dipalmitoylphosphatidylcholine (DPPC) with phase transition temperature of about 41^o^C, lysolipid of monostearoylphosphatidylcholine (MSPC) which enhances the thermosensitive capability, and poly (ethylene glycol) 2000-distearoylphosphatidylethanolamine (DSPE-PEG 2000) —at a molar ratio of 90: 10: 4, has been successfully developed. The permeability of this liposome can be greatly enhanced when the temperature reaches 40-42^o^C, resulting in rapid release of the encapsulated DOX in the target 2, 10, 11. ThermoDox has been demonstrated to be superior to the Doxil formulation in releasing encapsulated DOX 12, 13 and has begun with hepatocellular carcinoma treatment in phase III clinical trials 10, 13. Although a significant tumor therapeutic effect has been observed 12, 13, plasma proteins in the blood often interact with liposomes via electrostatic, hydrophobic, and van der Waals interactions, resulting in lysolipid dissociation from the carrier, causing about 50% of encapsulated DOX being released within 1h 14, 15. Later, another thermo-responsive liposomal formulation without lysolipid was developed, which consisted of DPPC, cholesterol, and DSPE-PEG 2000 at a molar ratio of 60: 40: 5. Its thermosensitive component relies on the core ammonium bicarbonate (ABC, NH_4_HCO_3_), which can not only greatly enhance the DOX encapsulation efficiency by the remote transmembrane gradient loading method, but can also decompose to generate CO_2_ bubbles when heated to 40^o^C and above. Permeable defects can thus be created in the lipid bilayer to induce a rapid DOX release and further locally increase the drug concentration, showing improved therapeutic results 16-18. However, encapsulated drugs or ABC leakage or burst release in these liposome systems may occur during storage or blood circulation, which would compromise the therapeutic efficacy and inevitably increase adverse side effects 19-21. Therefore, it is still highly challenging and desirable to construct drug delivery systems (DDSs) capable of stably retaining a drug during blood circulation while releasing the drug precisely and in a controlled manner in the target tissues.

To resolve the instability problem of liposomes, we recently developed a liposomal nanohybrid formulation called cerasome 22-28. It has a bilayer nanostructure similar to liposomes and has an atomic layer of polyorganosiloxane surface resembling silica, which combines the advantages of both liposomes and silica, showing good biocompatibility and high morphological stability 22-28. When anticancer drugs are loaded into such a system, premature release can be greatly avoided, plus prolonged blood circulation time, and more chemotherapeutic drugs can be delivered to the tumor while having fewer side effects on healthy tissues 22-28. However, cancer drug resistance may occur due to the slow drug release rate from this highly stable system. Therefore, a blood-stable cerasome with a controlled drug release property may address this issue, but that is still a great challenge since it is difficult to simultaneously achieve good stability and effective controlled drug release in the desired tissues 29.

In order to develop a controlled drug delivery system into tumor cells, many multifunctional nanoparticles with environmentally sensitive (pH 30-35, temperature 16, 17, 29, 36, enzymes 37, etc.) capabilities have been developed 38-41. Among these, the NIR mediated photothermal effect has showed great promise in nanomedicine, which should be attributed to the non-invasiveness, deep tissue penetration, and spatiotemporal controlled behavior of NIR light 42-48. Photo induced hyperthermia alone can directly cook and kill cancer cells, while NIR photothermal-triggered release of chemotherapeutic agents upon NIR irradiation can well realize the combinational photothermal therapy and chemotherapy in a precisely controllable manner, achieving a more efficient tumor killing effect 49-55. Many conventional NIR resonant nanomaterials such as gold nanostructure and carbon nanomaterials have been developed in recent years 56-64. However, these systems still face some challenges: 1) The non-biodegradability of these inorganic materials causes concerns of long-term toxicity. 2) They lack a probe for monitoring drug distribution in a real-time and a non-invasive manner, which plays an important role in determining the time and site of performing NIR irradiation. 3) Drugs loaded with a non-covalent force often lead to undesirable premature release in circulation. In contrast, biodegradable NIR dyes such as indocyanine green (ICG) 51, cypate 56, IR780 57, and DiR 47, 65, 66 with excellent photothermal conversion efficiency and real-time fluorescence imaging capability are more promising candidates for photothermal therapy. However, when performing direct systematic administration, seriously premature release still occurs when dyes are encapsulated in the liposomes, which often restricts their application in photothermal therapy 67, 68. Therefore, the combination of NIR fluorescence dyes with a highly stable cerasome may construct a novel drug delivery system to improve therapeutic outcomes.

**Scheme 1:**
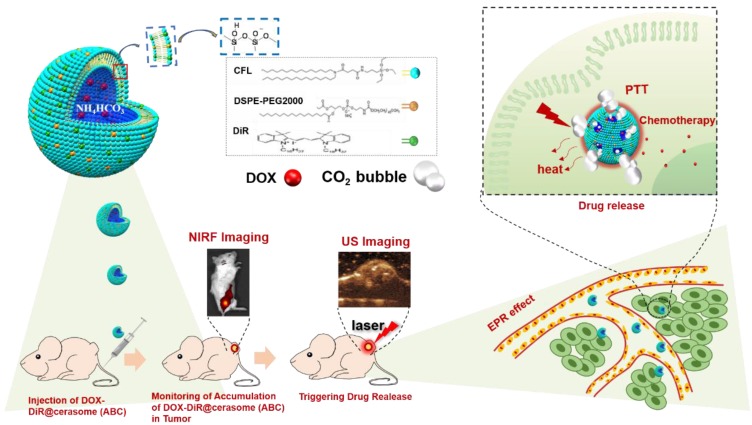
Schematic illustrations showing the structure of thermo-responsive bubble-generating cerasome system and its process of controlled drug release, NIR florescence (NIRF) imaging and ultrasound (US) imaging guided chemo-photothermal Therapy. DiR was assembled in the hydrophobic membrane, while ammonium bicarbonate (ABC) and chemotherapeutic agent DOX were encapsulated in the hydrophilic inner cavity. DiR can be used to guide laser irradiation time and site for achieving precise hyperthermia in tumor site. Under the 760nm laser irradiation, strong thermal energy generated from DiR resulted in decomposed ABC which generated CO_2_ bubbles, resulting in triggering of rapid drug release.

In this work, a photo and thermal sensitive cerasome was constructed by co-assembly of a cerasome-forming lipid (CFL) with a photothermal agent of DiR, while encapsulating both hyperthermia-responsive ABC salt and chemotherapeutic agent DOX, affording a bubble-generating DOX-DiR@cerasome (ABC) to develop a NIR photothermal-responsive drug release for combinational tumor therapy (Scheme 1). In this system, the highly stable cerasome can well retain drugs and DiR inside this carrier at body temperature, attributed to its silicate framework surface, greatly avoiding any undesirable premature release during blood circulation, thus reducing side effects on healthy tissues and organs. Furthermore, the NIR fluorescence dye of DiR can be used to guide laser irradiation for achieving precise hyperthermia in tumor sites, which would further decompose ABC locally to generate CO_2_ bubbles, creating permeable channels to facilitate rapid DOX release through the cerasomal bilayer. The generated CO_2_ bubbles could be easily detected by ultrasound imaging because of their hyper echogenicity, providing an indirect way for real-time monitoring of the drug release process. Such a strategy was very important for achieving localized drug release at tumor sites with less premature release, combined with a DiR induced photothermal effect, holding a great promise for cancer theranostics.

## 2. Results and Discussion

### 2.1. Preparation and characterization of DOX-DiR@cerasome (ABC)

In this study, to achieve both high stability and efficient photothermal conversion, a lipid formulation of CFL with silicate precursor, DiR, and DSPE-PEG 2000 with a molar ratio of 10 : 2.7 : 1 were selected based on our previous studies 47. Then, these lipids were dissolved in ethanol and injected into an aqueous saturated ABC solution (2.7 M) to start the self-assembly of the cerasomal bilayer, followed by in-situ surface silanol groups condensation (Si-OCH_2_CH_3_ + H_2_O→Si-OH + CH_3_CH_2_OH, followed by 2Si-OH→Si-O-Si + H_2_O), affording a nanohybrid of DiR@cerasome (ABC) with a surface silicate framework (Scheme 1). In order to maximize the generation of CO_2_ bubbles, the concentration of ABC solution used here was at its saturation concentration 16, 69. As a lipophilic molecule, DiR with two long carbon chains could easily be integrated into the hydrophobic cerasome membrane via hydrophobic interactions with an encapsulation efficiency above 98% 70. DOX was subsequently loaded into the DiR@cerasome (ABC) by a remote active-loading technique based on the transmembrane ABC gradient to obtain the final DOX-DiR@cerasome (ABC) (Figure S1) 16. The control cerasome formulated in citric acid buffer (CA) (DOX-DiR@cerasome (CA)) and conventional liposomes (DOX-DiR@liposome (ABC)) were both prepared by the same procedure. The characteristics data of these three nanoparticles were summarized in Table 1. DOX-DiR@cerasome (CA) and DOX-DiR@cerasome (ABC) showed similar particle sizes, smaller than DOX-DiR@liposome (ABC). Both of them showed similar polydispersity and zeta potential. Similar DOX encapsulation efficiency (> 90%) and loading content (~ 10%) were also achieved by the remote loading method, which were beneficial for subsequent comparison.

**Figure 1:**
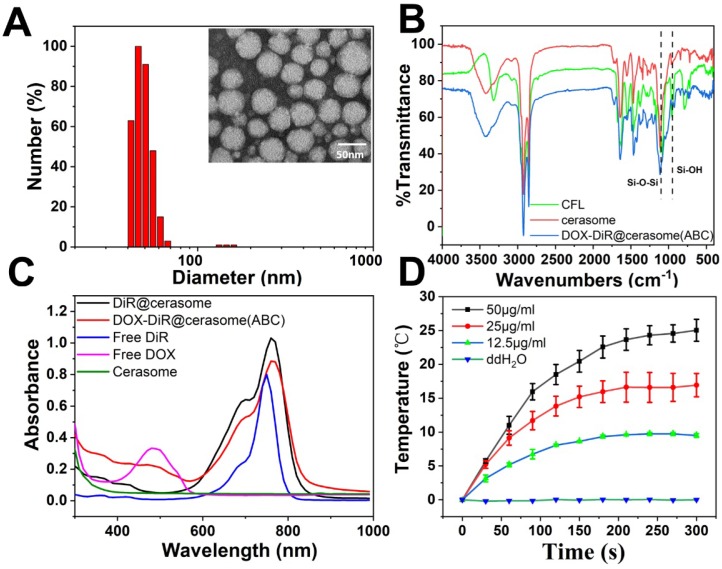
** A)** Size distributions and TEM micrograph of DOX-DiR@cerasome (ABC). **B)** FT-IR spectroscopy of CFL, cerasome and DOX-DiR@cerasome (ABC); **C)** UV-vis-NIR absorbance spectra of DOX-DiR@cerasome (ABC), DiR@cerasome, Free DiR, Free DOX, and cerasome; **D)** Photothermal elevation curve of DOX-DiR@cerasome (ABC) at different concentrations when irradiated with 760 nm laser for 5 min (1 W/cm^2^). Data shown as means ± SD (n = 3).

As shown in Figure 1A, DOX-DiR@cerasome (ABC) exhibited spherical morphology with uniform size distribution (39.7±8.4 nm), consistent with the dynamic light-scattering (DLS) measurements, showing a remarkably narrow distribution (45.9 ± 4.3nm). The content of NH_4_HCO_3_ in one cerasome with a diameter of ~40nm was calculated to be about 7.2 × 10^-12^μg. The zeta potential of DOX-DiR@cerasome (ABC) was negatively charged (-41.87 ± 0.24 mV) attributed to the surface silanol groups on the DOX-DiR@cerasome (ABC), making the resulting nanoparticles disperse well in different media (Figure S2). The morphological stability of cerasome is inherent for excellent *in vivo* therapeutic application. A simple surfactant solubilization method was used to compare the morphological stability of the cerasome and liposome in an aqueous media 29, 71. As shown in Figure S3A, the conventional liposomes of DOX-DiR@liposome (ABC) when mixed with the nearly 5 equivalent molar ratios of nonionic surfactant Triton X-100 (TX-100) resulted in the drastic change in diameter, indicating a collapse of the liposome. In contrast, the DOX-DiR@cerasome (ABC) were significantly resistant against the TX-100, indicating relatively higher stability as compared to the conventional liposomes at similar experimental condition. The higher stability of cerasome could be mainly due to the formation of siloxane networks surrounding the cerasome surface.

The silicate framework on the DOX-DiR@cerasome (ABC) surface was further examined by Fourier transform infrared (FT-IR) spectroscopy 72, showing absorption peaks at 1100cm^-1^ and 950cm^-1^, belonging to Si-O-Si and Si-OH groups respectively. In addition, the intensity of the former peak was significantly higher than the latter one, further confirming the dense Si-O-Si network formation on the surface of DOX-DiR@cerasome (ABC) (Figure 1B). The presence of Si in the DOX-DiR@cerasome (ABC) was also confirmed by SEM-EDS analysis (Figure S5B). The absorption spectra of DOX-DiR@cerasome (ABC) showed two characteristic peaks of DOX and DiR at about 488nm and 760nm (Figure 1C), consistent with the absorption spectra of free DOX and free DiR, respectively, indicating the successful loading of DOX and DiR. In contrast to free DiR (748 nm in ethanol), the slightly red shift in the absorption spectra of DiR after the formation of DOX-DiR@cerasome (ABC) may be due to its aggregation in the encapsulated state. To investigate the photothermal effect of DOX-DiR@cerasome (ABC), the aqueous samples with different concentrations were irradiated by 760nm laser for 5min (Figure 1D), exhibiting temperature elevation of 9.7°C, 16.9°C, and 25.5°C for different concentrations of 12.5μg/mL, 25μg/mL, and 50μg/mL, respectively. In contrast, the deionized water group showed no significant temperature change, further confirming the high photothermal conversion capability of DOX-DiR@cerasome (ABC), showing a great potential for cancer chemo-photothermol therapy.

### 2.2. Bubble-generating capability and *in vitro* controlled drug release

**Figure 2:**
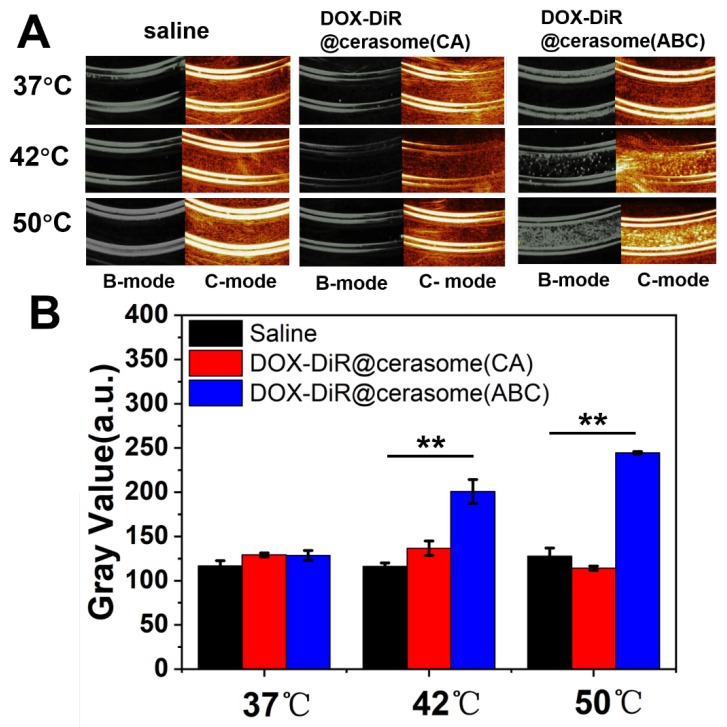
** A)** *In vitro* US imaging of saline, DOX-DiR@cerasome (CA) and DOX-DiR@cerasome (ABC) in a test tube at different temperature; **B)** Quantitative analysis of US imaging signal in different groups. Data shown as mean ± SD (n = 3), **P < 0.01.

ABC encapsulated in the core of DOX-DiR@cerasome (ABC) can be decomposed into water, ammonia, and CO_2_ bubbles, which can not only create permeable channels in the bilayer membranes to enable drug release, but also provide a way for monitoring the drug release process since the CO_2_ bubbles are hyperechogenic and can serve as an enhancer for ultrasound imaging 16, 17, 73. To investigate the bubble-generating potential *in vitro*, DOX-DiR@cerasome (ABC) solution was placed in a latex tube, then immersed into a water bath at different temperatures, and the generation of CO_2_ bubbles was evaluated by an ultrasound imaging system. As shown in Figure 2A, there was no ultrasound contrast effect in the latex tube with DOX-DiR@cerasome (ABC) at 37°C. In contrast, a significant ultrasound imaging signal was observed when the temperature was elevated to 42°C, which should be attributed to the generated CO_2_ bubbles. In addition, the higher of the temperatures (50°C) induced stronger ultrasound imaging signal intensity, indicating more CO_2_ bubbles were generated. The imaging stability of the generated CO_2_ bubbles was further investigated, demonstrating that obvious ultrasound imaging signal can last about 10min (Figure S4). Meanwhile, for the control groups of saline and DOX-DiR@cerasome (CA), no ultrasound contrast effect was detected at all tested temperatures, indicating no bubbles were generated in these systems. The ultrasound imaging signal intensity was further quantitatively analyzed using *ImageJ Analysis System* (Figure 2B). The relative signal intensity of DOX-DiR@cerasome (ABC) was much higher than that of saline and DOX-DiR@cerasome (CA) at 42°C and 50°C. These studies demonstrated that the ABC decomposition in the DOX-DiR@cerasome (ABC) could enable immediate CO_2_ bubble formation through thermal activation, which was very important for stimulating and monitoring drug release.

**Figure 3:**
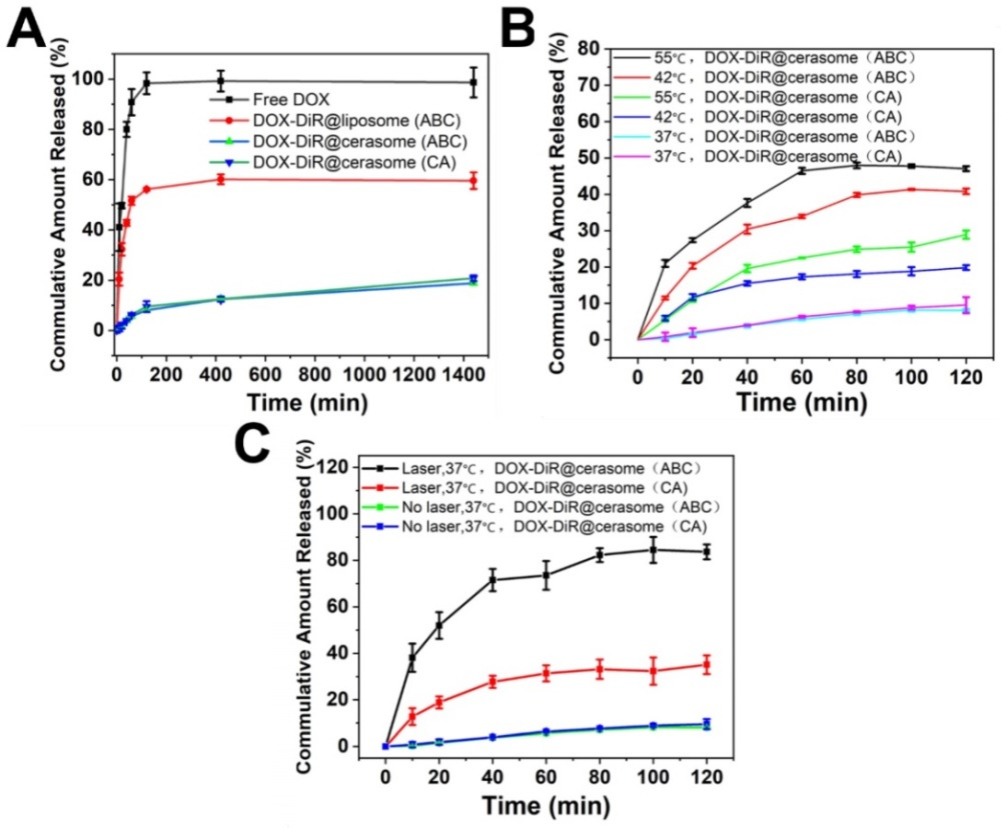
*In vitro* release behavior of DOX from the vesicular composition at different temperature.** A)** DOX release profiles from DOX-DiR@liposome (ABC), DOX-DiR@cerasome (CA) and DOX- DiR@cerasome (ABC) in 37 °C water bath for 24 h, free DOX was used as a control;** B**) DOX release profiles from DOX-DiR@cerasome (CA) and DOX-DiR@cerasome (ABC) in 37 °C, 42 °C and 55 °C water bath for 120 min, respectively; **C)** DOX release profiles from DOX-DiR@cerasome (CA) and DOX-DiR@cerasome (ABC) in 37 °C water bath for 2h with or without laser irradiation for 20 min. Data shown as mean ± SD (n = 3).

An ideal stimuli-sensitive drug delivery system should be stable enough but also rapidly respond to certain stimuli. Thus, the drug release profiles of DOX-DiR@cerasome (ABC) with or without heating were performed to investigate the influence of heat or generated bubbles on the drug release. As shown in Figure 3A, both DOX-DiR@cerasome (ABC) and DOX-DiR@cerasome (CA) were stable at 37°C in PBS (pH 7.4), showing a cumulative DOX release of only about 20% over 24h, while conventional DOX-DiR@liposome (ABC) release about 60% under the same conditions, indicating the higher stability of the cerasome system attributed to its silicate framework surface. The release of DOX was significantly accelerated when the samples were placed at constant mild hyperthermia of 42°C and 55°C, showing 19.9% and 28.9% DOX release for DOX-DiR@cerasome (CA) in 120min, and 41.3% and 48% DOX release for DOX-DiR@cerasome (ABC), greatly higher than the data of about 8.0% DOX release at 37°C (Figure 3B). Therefore, without heating, the cerasome system was stable, and with hyperthermia, the permeability of the bilayer membranes was increased and CO_2_ bubbles generated by the heat induced ABC decomposition created further drug release channels, thus accelerating drug release even more. To further evaluate the NIR-triggered DOX release, laser irradiation (760nm, 1W/cm^2^, 20min) was applied to stimulate the drug release, exhibiting significantly enhanced DOX release of about 83.7% in 120min for DOX-DiR@cerasome (ABC), which was about 10 times higher than DOX-DiR@cerasome (ABC) without NIR laser treatment (~8.0%) (Figure 3C). The likely mechanism should be because DiR can transfer NIR energy to thermal energy, improving the permeability of the cerasome membrane and leading to the decomposition of ABC to generate CO_2_ bubbles, subsequently triggering rapid DOX release. In contrast, the DOX release was much slower from DOX-DiR@cerasome (CA), which should be due to the lack of bubble generation. To investigate the stability of DOX-DiR@cerasome (ABC) under NIR laser irradiation, TEM images of the tested cerasome after laser irradiation were investigated. As shown in Figure S5A, DOX-DiR@cerasome (ABC) still maintained its morphology integrity, indicating its stability under NIR laser irradiation, which should be attributed to its surface siloxane framework (Figure 1B & Figure S5B) 22, 29, 72. Overall, the good stability of DOX-DiR@cerasome (ABC), together with its rapidly accelerated drug release under hyperthermia, made it possible for effective anticancer drug delivery with reduced undesirable premature leakage and on-demand site specific release, thus enhancing chemo-photothermal combination therapy.

### 2.3. *In vitro* cellular uptake and cellular drug release

**Figure 4:**
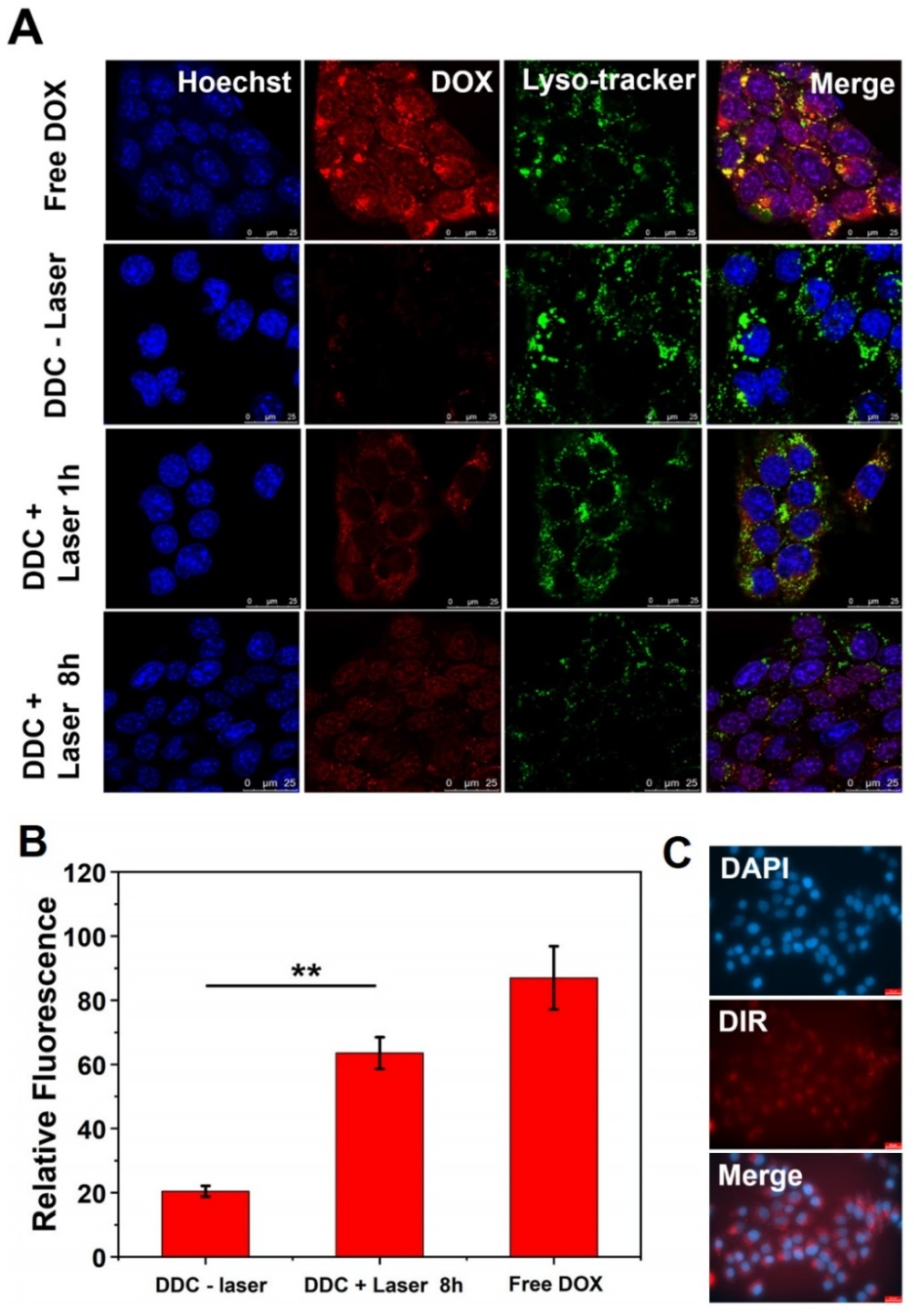
*In vitro* cellular uptake of DOX-DiR@cerasome (ABC). **A)** Representative CLSM fluorescence images of 4T1 tumor cell internalization of free DOX and DOX-DiR@cerasome (ABC) for 4h without or with 760 nm laser irradiation, followed by incubation for another 1h or 8h, scale bar = 25 μm. **B)** Relative fluorescence intensities of DOX in the 4T1 cell nucleus in different groups were semi-quantified. Data were presented as mean ± SD (n = 5). **P < 0.01. **C)** Fluorescence microscope images of DiR in 4T1 cells treated with DOX-DiR@cerasome (ABC) after incubation for 4 h. Scale bar = 25 μm. (DDC: DOX-DiR@cerasome (ABC)).

The cellular uptake and distribution of DOX-DiR@cerasome (ABC) in 4T1 cells were investigated by confocal laser scanning microscope (CLSM) and fluorescence microscope, that showed a homogeneously aggregated fluorescence of DOX and DiR mainly distributed in the cytoplasm (Figure 4A&C). Without laser stimulus, DOX was restrained in the aqueous core of DOX-DiR@cerasome (ABC) with less premature release, and the DOX fluorescence was self-quenched in an aggregated state at different incubation time (Figure 4A and Figure S6), indicating stability of this system at 37°C. However, it was difficult for DOX-DiR@cerasome (ABC) with a large particle size to get into the nuclei 46, 74. Therefore, controlled drug release in the cell was further investigated. NIR-laser irradiation was then performed on the DOX-DiR@cerasome (ABC) treated cells for 5min (760nm, 1W/cm^2^). Then, the cells underwent another 1h incubation at 37°C. As shown in Figure 4A, a significantly stronger DOX fluorescence signal homogenously distributed in the cytoplasm was observed, indicating the DOX release from DOX-DiR@cerasome (ABC) and DOX fluorescence was obviously recovered. When increasing the incubation time for 8h, DOX fluorescence was observed to be homogenously distributed in both the cell cytoplasm and nuclei, demonstrating effective NIR stimulated drug release and followed by subsequent drug translocation into the nuclei. The semi-quantitative analysis revealed there was 3.1-fold higher DOX fluorescence intensity in the nuclei after these cells received laser irradiation and incubated for 8h (Figure 4B), further verifying the NIR photothermal-triggered drug release from the highly stable DOX-DiR@cerasome (ABC), consistent with the extracellular drug release results (Figure 3C). It was worth mentioning that the final distribution of DOX in the nuclei (Figure 4A) and DiR in the cytoplasm (Figure 4C) showed that DOX-DiR@cerasome (ABC) had a promising chemotherapeutic and photothermal therapeutic effect.

### 2.4. *In vitro* photothermal cytotoxicity of DiR@cerasome

**Figure 5:**
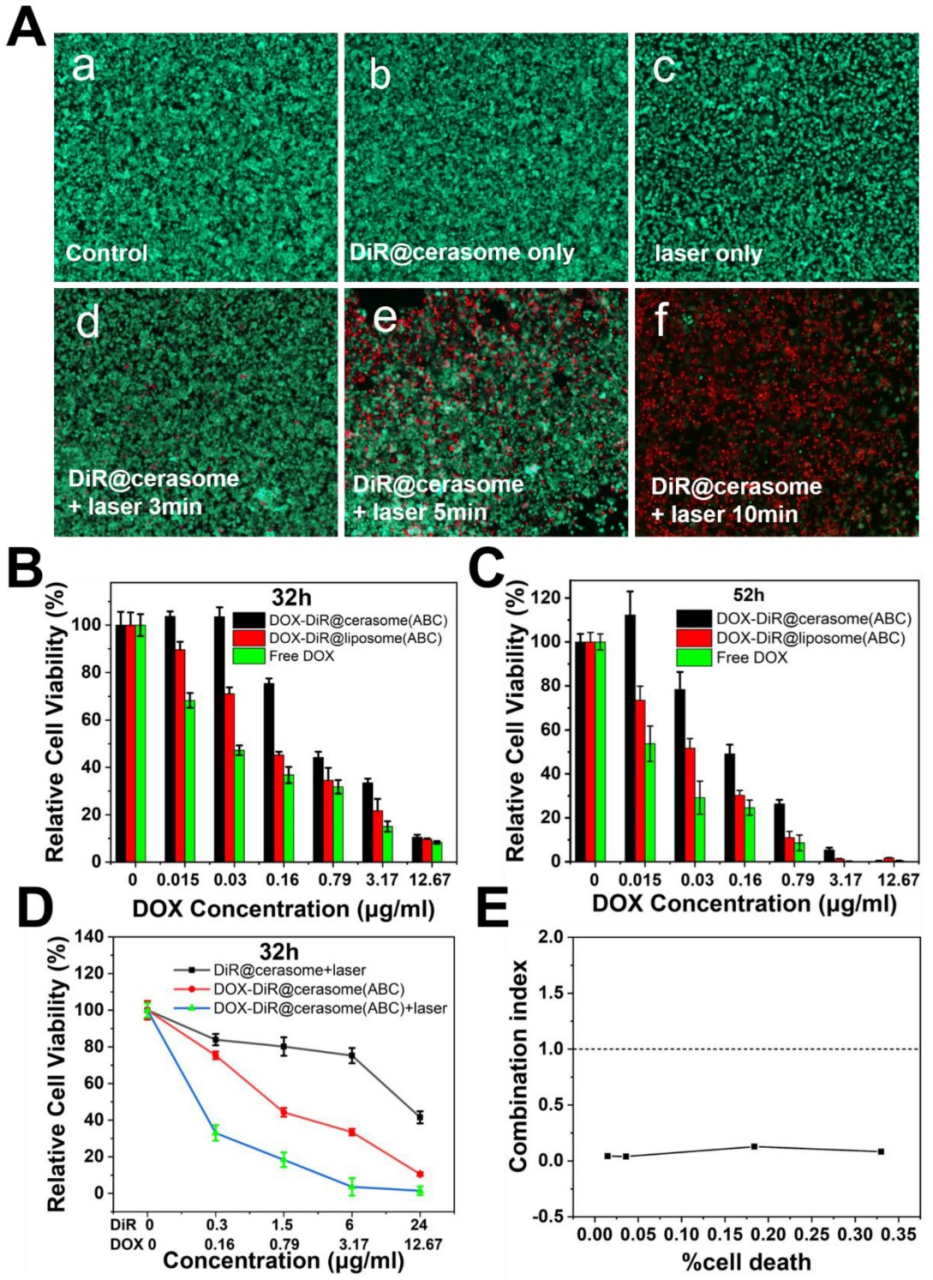
**A)** Photothermal destruction of 4T1 cells. (a) PBS; (b) DiR@cerasome only; (c) NIR laser (760 nm, 1W/cm^2^) only; (d) DiR@cerasome combined NIR laser for 3 min; (e) DiR@cerasome combined NIR laser for 5 min; (f) DiR@cerasome combined NIR laser for 10 min. **B)** *In vitro* chemotherapy and synergetic chemo-photothermal therapy of DOX-DiR@cerasome (ABC) in 4T1 cells after different treatments for 32h. **C)** *In vitro* chemotherapy and synergetic chemo-photothermal therapy of DOX-DiR@cerasome (ABC) in 4T1 cells after different treatments for 52h, DOX-DiR@liposome (ABC) and free DOX were used as controls. **D)** The cell viability of 4T1 cells after different treatments for 32h: DiR@cerasome + laser, DOX-DiR@cerasome (ABC), DOX- DiR@cerasome (ABC) + laser. **E)** Combination index (CI) of chemo- and photothermal dual therapy.

To avoid unnecessary damage to healthy tissues in photothermal therapy, biocompatibility of DiR@cerasome and safety parameters of the laser were first investigated in cells by MTT assay. As Figure S7A & S7B in the supporting information showed, DiR@cerasome exhibited little cytotoxicity to both HUVEC cells and 4T1 cells, with more than 90% of cells remaining viable when the DiR@cerasome concentration increased to 384μg/mL (DiR concentration), demonstrating its excellent biocompatibility. Similarly, the 4T1 cells showed high cell viability when treated with a 760nm laser at different power densities (0-1.5W/cm^2^) and for different irradiation times (0-20min), exhibiting excellent biosafety (Figure S7C & S7D). To further evaluate the therapeutic function of DiR, DiR@cerasome (DiR:40μg/mL) without DOX loading was then incubated with 4T1 cells for 4h, followed by NIR light exposure (760nm, 1W/cm^2^) for different durations. The cells treated with or without laser for different time intervals were further analyzed using calcein acetoxymethylester (calcein-AM) and propidium iodide (PI) staining agents. As shown in Figure 5A, the cells treated with phosphate-buffered saline (PBS) (Figure 5Aa), or only laser (Figure 5Ab), or only DiR@cerasome (Figure 5Ac) showed no obvious change in cell viability and density, consistent with the results shown in Figure S7. When treated with both DiR@cerasome and 3 min of laser irradiation (Figure 5Ad), the temperature increased to about 42 ℃ within 3min of laser irradiation, which was not high and long enough to kill the tumor cells, resulting in no significant cell death (Figure S8). In contrast, cell death could be observed when the irradiation time increased to 5min (Figure 5Ae). Further prolonging the irradiation time to 10min resulted in nearly all cells dying in the laser exposure area. These results indicated that the combination of DiR@cerasome and laser could significantly induce localized cellular death in a time-dependent manner.

### 2.5. *In vitro* chemotherapy and chemo-photothermal treatments

The *in vitro* chemotherapeutic effect of DOX-DiR@cerasome (ABC) was analyzed by MTT assay. 4T1 cells were incubated with DOX-DiR@cerasome (ABC), DOX-DiR@liposome (ABC) or free DOX for 32h and 52h, respectively. As shown in Figure 5B & 5C, all groups exhibited cytotoxicity in a dose and time-dependent manner. The free DOX treated group showed the strongest cytotoxicity, mainly because free DOX was small molecules, easily entering cells and generating strong toxicity. When DOX was encapsulated into liposomes, which needed time to release drugs, it exhibited lower anti-tumor activities than free DOX. DOX-DiR@cerasome (ABC) displayed the lowest cytotoxicity due to its high stability, resulting in the slowest drug release in the same period of time.

For investigating the therapeutic effect of chemo-photothermal combined treatments, cells treated with DOX-DiR@cerasome (ABC) were incubated for 4h and irradiated with 760nm laser (1W/cm^2^, 10 min), then followed by another incubation for 28h. Groups of DiR@cerasome + laser, and DOX-DiR@cerasome (ABC) without laser irradiation were tested for comparison. As shown in Figure 5D, all groups exhibited obvious cytotoxicity to the 4T1 cells in a concentration dependent manner. After treatment with the DiR@cerasome (containing 6μg/mL DiR) in combination with laser irradiation, the cell viability was evaluated to be 75.3 ± 2.1%, while DOX-DiR@cerasome (ABC) (containing 3.17μg/mL DOX and 6μg/mL DiR) treated group showed much more toxicity to the cells with viability of 33.5 ± 1.8 %. In stark contrast, when treated with both DOX-DiR@cerasome (ABC) and laser, the cell viability was significantly decreased to 3.6 ± 4.8%, showing the best killing capability. These data indicated the great synergistic anticancer activity of chemo-photothermal therapy of DOX-DiR@cerasome (ABC). Combination index (CI) was further calculated to analyze the combination effect of chemo- and photothermal dual therapy mediated by DOX-DiR@cerasome (ABC). Figure 5E showed the CI values of 4T1 cells plotted against the therapy effect levels at 32h incubation after therapy. All data points were below the line of CI = 1, confirming the great synergistic anticancer activity of chemo-photothermal therapy. The mechanism of this synergistic effect should be due to the enhanced release of DOX from the bubble-generating DOX-DiR@cerasome (ABC) by the local hyperthermia and subsequent increased cytotoxicity to the tumor cells, with cells exposed to DOX showing increasing heat sensitivity 75, 76. Therefore, the cytotoxic effect of DOX-DiR@cerasome (ABC) on tumor cells could be greatly enhanced by the NIR light stimulated chemo-photothermal therapy. However, it should be noted that the ABC-containing liposomes without drug loading had been investigated to induce cell necrosis through a bubble mediated transient cavitation by thermal activation^73^, thus the great synergistic anticancer activity of DOX-DiR@cerasome (ABC) should be due to the combination of chemotherapy, photothermal therapy, and bubble mediated transient cavitation.

### 2.6. *In vivo* near-infrared fluorescence imaging and ultrasound imaging

**Figure 6:**
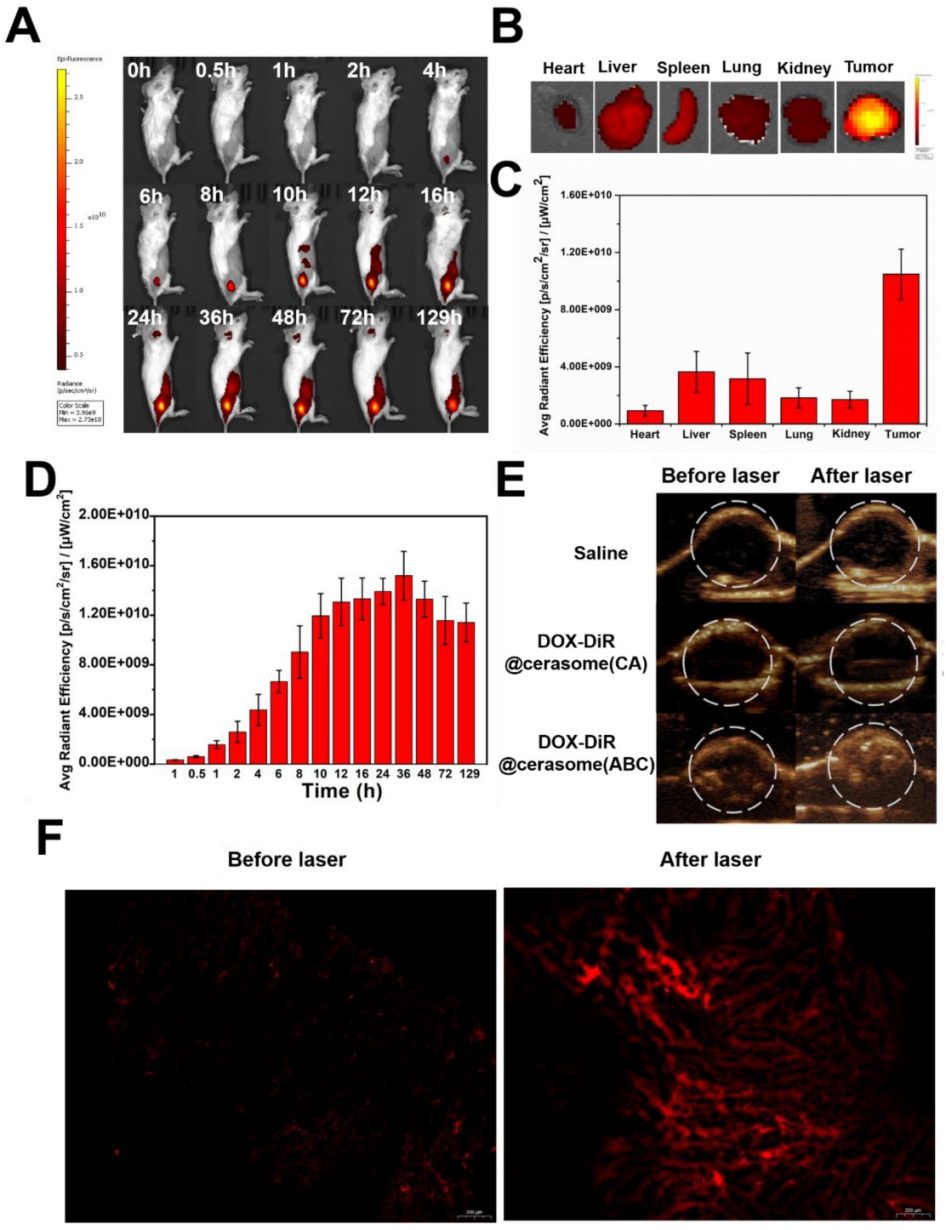
*In vivo* imaging and biodistribution of 4T1 tumor-bearing mice. **A)** Whole body NIR fluorescence images at different time after IV injection of DOX-DiR@cerasome (ABC). **B)** Ex vivo NIR fluorescence images of major organs and tumor at 129h post-injection. **C)** The quantification of fluorescence intensities of major organs and tumor. Data were presented as mean ± SD (n = 3). **D)** The quantification of fluorescence intensity of tumor obtained after tail vein injection of DOX-DiR@cerasome (ABC). **E)** *In vivo* ultrasound imaging of 4T1 tumor after IV injected with saline, DOX-DiR@cerasome (CA) and DOX-DiR@cerasome (ABC) before and after NIR laser irradiation at 36h post-injection.** F)** Fluorescent images of DOX of the frozen tumor sections following IV injection of the DOX-DiR@cerasome (ABC) before and after NIR laser irradiation at 36h post-injection.

The *in vitro* results encouraged us to further investigate the *in vivo* behavior of DOX-DiR@cerasome (ABC). After intravenous (IV) administration to 4T1 tumor-bearing mice, the *in vivo* distribution and tumor accumulation of DOX-DiR@cerasome (ABC) were evaluated by both fluorescence imaging originating from the intrinsic fluorescence of DiR and ultrasound imaging derived from the bubbles decomposed by ABC. As shown in Figure 6A, the DiR fluorescence at the tumor site gradually increased during the first 10h and reached a maximum at 36h post injection (Figure 6A & 6D), showing effective passive tumor accumulation through the enhanced permeability and retention (EPR) effect. In addition, this fluorescence imaging can last for more than 5 days, with a little fluorescence decrease at 129h post injection. Such longer fluorescence imaging ability was extremely beneficial for guiding laser to the tumor site and monitoring tumor homing properties of particles in real time (Figure 6D). The distribution of DOX-DiR@cerasome (ABC) in the main organs and tumors was then investigated by detecting the excised tissues' fluorescence at 129h (Figure 6B). The tumor fluorescence was much more obvious than the other main organs, further confirming the remarkable tumor accumulation property of this nanosystem (Figure 6B&C). To investigate the blood circulation dynamics of the drug carrier, a fluorescent probe of porphyrin-grafted lipid (PGL) 77-80 was used to label DOX-DiR@cerasome (ABC) and then the blood fluorescence intensity of PGL was investigated. As shown in Figure S9, the blood PGL signals decreased to ~52.9% at 4h post injection and further reduced to only ~7.4% at 24h. The elimination half-life (t_1/2_) of DOX-DiR@cerasome (ABC) was calculated to be 5.06 ± 0.39 h.

When DOX-DiR@cerasome (ABC) reached the tumor at 36h post-injection, NIR light irradiation guided by fluorescence imaging was performed to transform light energy into heat as mediated by the DiR dyes on the particles. The generated heat promoted the decomposition of ABC to produce bubbles in the carrier, thus enabling rapid drug release and efficiently killing the tumor cells. The generated CO_2_ bubbles were then detected by ultrasound imaging. As shown in Figure 6E, all groups showed no ultrasound contrast enhancement without laser irradiation, indicating no bubble generation. In contrast, significantly positive contrast enhancement of the tumor was observed for the DOX-DiR@cerasome (ABC) + laser treated group, while the tumors of mice treated with saline + laser and DOX-DiR@cerasome (CA) + laser showed no ultrasound contrast effect. In addition, the ultrasound contrast enhancement was maintained in a stable state after prolonging the imaging time to 5min, which should be long enough for the accurate diagnosis in clinic application (Figure S10). Then, tumor sections were examined histologically to investigate the intratumoral release of DOX from DOX-DiR@cerasome (ABC). As shown in Figure 6F, minimal fluorescence signals existed in the tumor section before laser irradiation, indicating the limited release of their encapsulated DOX. After exposure to laser, a significantly stronger DOX fluorescence signal was observed, indicating the DOX release from DOX-DiR@cerasome (ABC) and DOX fluorescence was recovered. These results provided an indirect proof that highly stable DOX-DiR@cerasome (ABC) can retain ABC and DOX encapsulated in the carrier during blood circulation, and thus maximized the enrichment and controlled the release of therapeutic drugs in tumor tissues. The sustained strong fluorescence of DOX-DiR@cerasome (ABC) together with its capability of ultrasound imaging enabled the real-time monitoring of the *in vivo* delivery and drug release process of this system 47, 66, 81, 82.

### 2.7. *In vivo* chemo-photothermal therapeutic assay

**Figure 7:**
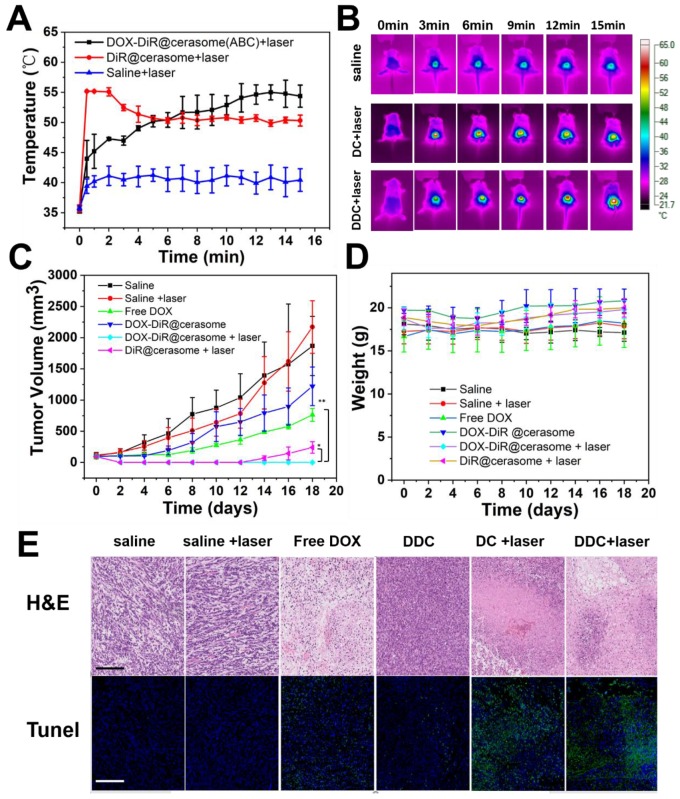
*In vivo* chemo-photothermal therapy of DOX-DiR@cerasome (ABC) on 4T1 tumor-bearing mice. **A)** The temperature change curves after IV injection of saline, DiR@cerasome, and DOX-DiR@cerasome(ABC) under NIR laser irradiation and **B)** *In vivo* IR thermal imaging. **C)** Tumor volume changes with increasing time. Error bars were based on seven mice in each group (*P<0.05, **P<0.01). **D)** Changes of body weight with increasing time. **E)** Histological analysis of tumor tissues with H&E staining and TUNEL assay after different treatments. Scale bar = 200 μm. (DC: DiR@cerasame; DDC: DOX-DiR@cerasame (ABC)).

The prominent properties of high stability, photothermal conversion, bubble generation induced triggered drug release and excellent tumor accumulation of DOX-DiR@cerasome (ABC) inspired us to further investigate its *in vivo* combined chemo-photothermal therapeutic effects on a 4T1 tumor model. Mice bearing tumors of ∼100 mm^3^ were randomly divided into six groups, including saline, saline + laser, free DOX, DiR@cerasome + laser, DOX-DiR@cerasome (ABC), and DOX-DiR@cerasome (ABC) + laser (DOX dose: 5mg/kg; DiR dose: 9.4 mg/kg). The laser irradiation time was selected as 36h post-injection, as it showed the best tumor accumulation of the cerasome, which was revealed by the *in vivo* fluorescence imaging (Figure 6A & 6D). Laser irradiation was then performed (760 nm, 15min, 1W/cm^2^), and the surface temperature of the tumor was monitored using an infra-red (IR) thermal camera. For those mice treated with DOX-DiR@cerasome (ABC) + laser or DiR@cerasome + laser, their tumor surface temperature showed a rapid increase to ≈55^o^C, while saline + laser treated mice showed minimal change under the same conditions (Figure 7A & 7B), suggesting the good tumor accumulation of these cerasome nanoparticles and effective photothermal conversion mediated by the DiR, which was very important to ablate the tumor tissue and facilitate the bubble generation from ABC to trigger the drug release and subsequent translocation into the tumor cell nucleus. Then, the tumor volume of each group was recorded for 18 days. As shown in Figure 7C, a sharp increase in tumor volume was clearly observed for the mice treated with saline only or saline + laser, changing from 100mm^3^ to approximately 1800mm^3^ (18-folds) or 2000 mm^3^ (20-folds) within 18 days, respectively. These results indicated that laser irradiation alone cannot cause potentially destructive effects. The chemotherapy treated groups of free DOX and DOX-DiR@cerasome (ABC) only achieved partial tumor inhibition, which should be due to the limited tumor accumulation of free DOX and the slow drug release rate of DOX-DiR@cerasome (ABC) without laser irradiation, respectively. In stark contrast, both the DiR@cerasome + laser and DOX-DiR@cerasome (ABC) + laser treated groups exhibited remarkably high tumor inhibition, with almost complete tumor eradication during the first 12-day assay period. However, tumor recurrence was observed at 14 days for the DiR@cerasome + laser treated group, while no tumor recurrence occurred for the DOX-DiR@cerasome (ABC) + laser treated groups within the entire experimental period, which should be attributed to the synergistic effect of DiR mediated photothermal therapy and DOX mediated chemotherapy. Upon laser irradiation, the DiR on the cerasome bilayer can efficiently convert light to heat, which would further decompose the ABC in the cerasome core to generate CO_2_ bubbles, resulting in rapid-release of DOX with a local high concentration to kill the tumor cells via chemotherapy. In addition, the DiR induced hyperthermia further evoked the apoptosis of tumor cells via thermal therapy, thus leading to the best therapeutic effects.

To further investigate the therapeutic mechanism, the histological analysis of tumor tissues by H&E staining and TUNEL assay after different treatments was carried out (Figure 7E). The saline and saline + laser group displayed an intact nucleus morphology, indicating negligible cell apoptosis or necrosis, suggesting safety of the NIR laser irradiation. Both the free DOX and DOX-DiR@cerasome (ABC) only mediating chemotherapy exhibited a little cell apoptosis or necrosis, which should be due to the low DOX accumulation of free DOX and the slow drug release of DOX-DiR@cerasome (ABC) at the tumor site. In contrast, based on the photothermal effect of DiR, both DOX-DiR@cerasome (ABC) + laser and DiR@cerasome + laser groups displayed extensive nuclear shrinkage and fragmentation. From the fluorescence TUNEL assay, the areas without cell damage exhibited only blue color for saline and saline + laser groups. In contrast, the free DOX and DOX-DiR@cerasome (ABC) groups showed slightly higher intensity of green fluorescence, while the tumor tissues treated with DOX-DiR@cerasome (ABC) + laser and DiR@cerasome + laser showed obviously more green fluorescence, indicating serious cellular apoptosis, consistent with the H&E staining results. The histology examination of the tissue near the tumor was carried out, showing that the skin tissue near the tumor maintained in a healthy state (Figure S11C). During the whole experimental process, the six groups of mice showed insignificant body weight loss (Figure 7D), indicating that no obvious toxicity was caused by all the treatments. These results further demonstrated that DOX-DiR@cerasome (ABC) with both functions of photothermal and bubble triggered chemotherapeutic effects could produce significant synergistic therapeutic effect, holding great promise in inhibiting tumor recurrence and maintaining long-term therapeutic efficacy.

For assessing the *in vivo* toxicity of DOX-DiR@cerasome (ABC) to healthy tissues, the major organs such as heart, liver, spleen, lung, and kidney of the DOX-DiR@cerasome (ABC) treated mice without laser treatment were collected at 3, 7 and 30 days post treatment and the histological examination was further performed. As shown in Figure S11A, neither noticeable organ damage nor inflammation could be observed from the hematoxylin and eosin (H&E) stained images of the above major organs. Meanwhile, all groups showed insignificant body weight loss (Figure S11B), exhibiting good biocompatibility of DOX-DiR@cerasome (ABC).

## 3. Experimental Section

### 3.1. Materials and reagents

The cerasome forming lipid (CFL) of N-[N-(3-triethoxysilyl) propylsuccinamoyl]-dihexadecylamine was synthesized according to the procedures in the literature 28, 83. DSPE-PEG 2000 was the product of *Lipoid* (Ludwigshafen, Germany). 1,1'-Dioctadecyl-3,3,3',3'-tetramethylindotricarbocyanine iodide (DiR) and DOX were obtained from *Biotium* and *TCI* (Shanghai, China), respectively. DiR solution was prepared by dissolving it into ethanol. Roswell Park Memorial Institute (RPMI) 1640 Medium, 3-[4,5-dimethylthiazol-2-yl]2,5-diphenyltetrazoliumbromide (MTT), phosphate-buffered saline (PBS) were the products of *Invitrogen*. Calcein-AM, propidium iodide (PI) were purchased from *Biorj*. Deionized water (DI water) was obtained by purification of house-distilled water with a *Milli-Q Gradient System*. All of the other reagents and solvents were used as received.

### 3.2. Fabrication of DOX-DiR@cerasome (ABC)

DOX-unloaded thermosensitive cerasome (DiR@cerasome) was prepared using an ethanol injection method. Briefly, the mixture of CFL, DSPE-PEG2000, and DiR with certain molar ratios (10:1:2.7) was co-dissolved in ethanol, followed by rapid dispersion into an aqueous NH_4_HCO_3_ solution (ABC, 2.7 M) with water bath ultrasound sonication at room temperature, obtaining a homogeneous and opalescent suspension. Afterwards, the suspension was further homogenized by probe sonication for 3 min in an ice-water bath (*Sonifier*, 10% output amplitude). Then, dialysis method was performed to remove the unencapsulated ABC with a dialysis bag (MWCO 8000-14000) for 1h at room temperature. DOX was then loaded into the DiR@cerasome using the transmembrane ammonium bicarbonate gradient loading procedure 16. Briefly, aqueous DOX solution (10mg/mL) was added to the DiR@cerasome suspension with water bath ultrasound sonication at room temperature, then the sample was placed in a 37°C isothermal shaker for 24 hours, which benefited the formation of the silicate network on the cerasome surface. The whole procedure was performed in the dark and the obtained DOX-DiR@cerasome (ABC) was stored at 4°C before use. DOX-DiR@liposome (ABC) with the same DiR molar percentage was prepared as a control, consisting of DSPC, DSPE-PEG-2000, cholesterol, DiR with molar ratio of 11:1.5:10:4.5. The DOX-DiR@cerasome (CA) was prepared in citric acid buffer (300mM, pH 4.0) (CA) instead of ABC according to the similar procedure. The encapsulation efficiency (EE) and drug loading content (DLC) were calculated according to the following equations: EE(%) = (Weight of encapsulated DOX)/(Initial weight of DOX)*100; DLC(%) = (Weight of encapsulated DOX )/(Total weight of DOX-DiR@cerasome)*100.

### 3.3. Characterization of DOX-DiR@cerasome (ABC)

The morphology of DOX-DiR@cerasome (ABC) was detected by transmission electron microscopy (TEM, JEM-1400). Briefly, a 300-mesh Formvar-coated copper grid was first immersed into the particle suspension for 10 min to obtain the TEM sample, followed by negative staining with freshly prepared uranyl acetate aqueous solution (2wt%, 5 min), then washed with distilled water twice and air-dried for imaging. Data of both particle size distribution and zeta potential were examined and analyzed in automatic mode with a 90Plus/BI-MAS dynamic light scattering (DLS) analyzer (*Brookhaven Instruments Co.*). Vesicle size was expressed as intensity mean diameter ± SD of values. The absorption spectra were obtained by a UV-vis spectrophotometer (*Varian 4000*).

Fourier transform infrared spectroscopy (FT-IR) was performed on *a PerkinElmer spectrophotometer* (USA). All samples were pressed using potassium bromide (KBr) before measurement. CFL compound was directly mixed with KBr powder, and then grinded in a mortar, mixed evenly until there was no obvious particle in the powder, and then pressed. For the cerasome, the aqueous solution was first freeze-dried into solid powder, and then followed a similar procedure. Scanning wavenumber range was 400-4000 cm^-1^ with resolution of 4 cm^-1^. For *in vitro* ultrasound imaging, 1 ml of saline, DOX-DiR@cerasome (CA) and DOX-DiR@cerasome (ABC) was injected into a latex tube, and harmonic images were captured by a clinical ultrasound system (VINNO70, *Feino Technology (Suzhou) Co., Ltd*.).

### 3.4. Photothermal effect and *in vitro* drug release

For detecting temperature elevation of the samples induced by NIR light irradiation, the DOX-DiR@cerasome (ABC) with different concentrations (0, 12.5, 25, and 50μg/mL) were placed into a quartz cuvette with final volume of 0.5mL, then light irradiation was performed using a NIR laser (760 nm, 1W/cm^2^, 5 min, *Optoelectronics Tech. Co., Ltd.,* China), and the solution temperature was measured every 30s by a digital thermometer.

The *in vitro* drug release experiment of DOX-DiR@cerasome (ABC) with or without laser irradiation was carried out by the dialysis method in triplicate with irradiation time of 20min from the initial time at a certain temperature. Briefly, 0.45mL of DOX-DiR@liposome (ABC), DOX-DiR@cerasome (CA), DOX-DiR@cerasome (ABC) containing 90μg DOX was added into the dialysis bag (MWCO 8000-14000) and submerged into 90mL phosphate-buffered saline containing 0.1% Tween 80 (PBS, pH 7.4) at 37°C, 42°C and 55°C under horizontal shaking at 150 rpm for different durations. At scheduled time intervals, released medium (1 mL) was taken out and fresh medium of equal volume was replenished. The DOX released amount was determined by fluorescence measurements with excitation at 480nm and emission at 590nm.

### 3.5. Cell culture

The murine mammary carcinoma 4T1 cells were purchased from *American Type Culture Collection*. The human umbilical vein endothelial cells (HUVEC) were provided by the Central Laboratory of Peking University Third Hospital. Both 4T1 cells and HUVEC cells were cultured in RPMI-1640 medium supplemented with 500U/mL penicillin, 10% fetal bovine serum (FBS), and 50μg/mL streptomycin at 37°C in a humidified atmosphere containing 5% CO_2_.

### 3.6. Cellular uptake of DOX-DiR@cerasome (ABC)

The 4T1 cells were cultured in a confocal dish (5 × 10^5^ cells) with 2mL complete 1640 medium and incubated for 24h, followed by replacing with 0.5mL of fresh medium containing DOX-DiR@cerasome (ABC) or Free DOX (DOX: 12.5 μg/mL). Afterwards, the cells were further incubated for another 4h. To evaluate the photothermal influence on drug release, cells treated with DOX-DiR@cerasome (ABC) were then irradiated by NIR light (760nm, 1W/cm^2^) for 5min, then continued to be cultured for 1h or 8h. The culture media were removed and washed with PBS and then re-cultured in fresh medium containing Lyso-tracker red 100 × 10 ^-9^ M for 30min. The cell nuclei were stained with Hoechst 33342. Finally, the cells were imaged by confocal laser scanning microscopy (CLSM, TCS-SP8,* Leica*).

### 3.7. *In vitro* photothermal, chemotherapy, chemo-photothermal therapy

To avoid unnecessary damage to healthy tissues during photothermal therapy, biocompatibility of DiR@cerasome and safety parameters of the laser were investigated in cells by MTT assay. Briefly, HUVEC cells and 4T1 cells were plated at a density of 3 x 10^3^ cells/well in 96-well plates at 37°C in a 5% CO_2_ atmosphere. Wells without cells were used as the blank control. At 24h, the medium was replaced with fresh medium containing DiR@cerasome with different concentrations of 24, 48, 96, 192, and 384μg/mL (DiR concentration) and incubated for another 72h, then mixed with the MTT solution (10μL, 5mg/mL). Following incubation at 37°C for 4h in the dark, the medium was then removed and the formazan crystals in each well were dissolved by adding 150μL DMSO, followed by measurement with a *Bio-Rad model 680* plate reader to obtain the absorbance data in each well at 570nm. To study the safety of the laser, the 4T1 cells were irradiated by 760nm laser for different durations (1W/cm^2^, 0-20min) and different power densities (0-1.5W/cm^2^, 10min), then cultured overnight, and cell viability was tested by MTT assay.

To further investigate the photothermal therapy induced cell cytotoxicity, 4T1 cells were incubated in 24-well plates overnight (2 × 10^5^ cells/well, 37°C, humidified atmosphere, 5% CO_2_). DiR@cerasome (0.5mL/well, 40μg/mL) was then added in and incubated for 4h, the 24-well plate was fixed on the iron stand and the laser illuminated the bottom of pates from below to top for 10min at the same spot. The diameter of the laser spot was about 0.7cm, similar to the diameter of a well in 96-well plate. The cells were irradiated by the NIR light (760nm, 1 W/cm^2^) for 0, 3, 5, and 10min, the photothermal effect of DiR@cerasome on cancer cells was examined by calcein acetoxymethyl ester (calcein-AM) and propidium iodide (PI) staining.

Cell cytotoxicity of chemotherapy and chemo-photothermal treatment was further investigated with 4T1 cells seeded in 96-well plates (5 × 10^3^ cells/well). After incubating overnight, the medium was removed and replaced by fresh media containing determined concentrations of free DOX, DOX-DiR@liposome (ABC), DOX-DiR@cerasome (ABC) and DiR@cerasome. For DiR@cerasome + laser and DOX-DiR@cerasome (ABC) + laser group, cells were irradiated with 760nm laser for 10min after 4h of incubation. After 32h or 52h of incubation, the medium was removed and the formazan crystals in each well were dissolved by 150μL DMSO. The absorbance at 570nm in each well was measured by a *Bio-Rad model 680* plate reader.

### 3.8. *In vivo* imaging and biodistribution of DOX-DiR@cerasome (ABC)

Female Balb/c mice (18-20g) were obtained from *Beijing Vital River Laboratory Animal Technology Co., Ltd.* The animal experimental protocols were approved by Peking University Institutional Animal Care and Use Committee (IACUC). NIR fluorescence imaging analysis was conducted to investigate the *in vivo* biodistribution of DOX-DiR@cerasome (ABC) on an *IVIS Imaging Spectrum System* fixed with *IVIS Living Imaging 3.0* software (*PerkinElmer*, λex = 740nm, λem = 800nm, exposure time = 0.1s). Firstly, tumor implantation was carried out by injecting 100μL of 4T1 cell suspension (5 × 10^6^ cells) in the right back of mice. When the tumor volume reached ∼100mm^3^, DOX-DiR@cerasome (ABC) (100μL, 0.487mg/mL DiR) was injected into the tumor-bearing mice via the tail vein, followed by NIR fluorescence imaging at predetermined time points.

To investigate the acoustic behavior of DOX-DiR@cerasome (ABC) after laser irradiation, ultrasound imaging was performed on female Balb/c tumor-bearing mice using a contrast-enhanced ultrasound imaging mode (VINNO70, *Feino Technology (Suzhou) Co., Ltd.*). The mice were divided into three groups, which were injected via tail vein with saline, DOX-DiR@cerasome (CA) and DOX-DiR@cerasome (ABC). At 36h after injection, the tumors were irradiated by the 760nm laser at 1W/cm^2^ for 10min, followed by ultrasound imaging observation. To observe fluorescence images of DOX in tumor sections, the mice were killed and their tumors were separated and frozen at -80^o^C, then cryo-sectioned in 5~7μm thicknesses. The frozen sections were digitally scanned (*Pannoramic MIDI*) and visualized by the software *Case Viewer.*

### 3.9. *In vivo* combined anticancer activity and histology analysis

For the tumor therapeutic experiment, 4T1 tumors bearing mice were randomly divided into six groups (n = 7). 4T1 cell suspension (100μL, 5×10^6^ cells) were injected into the right back of mice before the experiment. After the tumor volume reached ~100mm^3^, mice were treated with saline, saline + laser, free DOX, DiR@cerasome + laser, DOX-DiR@cerasome (ABC), and DOX-DiR@cerasome (ABC) + laser, respectively. At 36h after injection, for the laser groups, tumors were irradiated by the 760nm laser at 1W/cm^2^ for 15min. An infra-red (IR) thermal camera was used to measure the temperature variation of the tumors. During the experiment, the tumor size and mice body weight were recorded at predetermined time. Tumor volume (V) was calculated according to the following formula: V (mm^3^) = 1/2 × length (mm) × width (mm)^2^. For determining the blood clearance of DOX-DiR@cerasome (ABC), healthy Balb/c mice were administrated with PGL labelled DOX-DiR@cerasome (ABC) via the tail vein. The PGL levels in mice plasma were determined by a multiplate microreader. Briefly, blood samples were collected at certain time intervals from the mice with a heparin-coated syringe. Then centrifugation (3000 g, 10 min) was performed to separate the plasma and the supernatant absorbance at 650 nm was examined to measure the PGL fluorescence intensity by a multiplate microreader (*Synergy, Bio Tek*). The elimination half-life (t_1/2_) was determined by a pharmacokinetic analysis using *Origin (9.1)* by fitting the curve with an ExpDec1 exponential function. At the end of different treatments, the mice were killed and the tumors were collected, fixed in 10% formalin, and embedded in paraffin, then TUNEL assay was conducted to measure the intratumoral late apoptosis and hematoxylin and eosin (H&E) staining was carried out for the apoptosis and necrosis evaluation. For toxicology evaluations, healthy Balb/c mice were administrated with DOX-DiR@cerasome (ABC) (5mg/kg DOX, 9.4mg/kg DiR), which was equal to the therapeutic dose. Major organs of liver, heart, spleen, lung and kidney were collected for investigation of the histopathology changes after H&E staining at 3, 7 and 30 days post injection.

### 3.10. Statistical analysis

Related data was presented as mean ± standard deviation (SD). The significance of the difference was tested by One-way analysis of variance (ANOVA), *P< 0.05 and **P< 0.01 were considered to be significant.

## 4. Conclusions

In this study, a highly stable cerasome-based drug delivery system was fabricated for NIR light triggered drug release through bubble generating, thus inducing combined chemo-photothermal therapy. By self-assembly with NIR dye of DiR and co-encapsulating bubble generating ABC and chemotherapeutic drug of DOX, we obtained a promising local triggering drug delivery system DOX-DiR@cerasome (ABC) with a silicate framework, which endowed the nanosystem with high stability during blood circulation, efficient tumor accumulation, and rapid drug release upon laser irradiation. Interestingly, the DiR fluorescence at the tumor site could last for more than 5 days, facilitating the *in vivo* real-time monitoring distribution and location of DOX-DiR@cerasome (ABC). Upon NIR irradiation guided by DiR fluorescence, significant bubble generation could be detected by ultrasound imaging, supplying an indirect way to monitor the drug release process. The CO_2_ bubbles created channels in the DOX-DiR@cerasome (ABC) bilayer, triggering rapid DOX release. In addition, the DiR on DOX-DiR@cerasome (ABC) showed high light-to-heat conversion in the tumor cell cytoplasm for effective photothermal therapy. Thus, the rapid thermal stimulated quick release of DOX initiated by efficient photothermal therapy both *in vitro* and *in vivo* together with dual modal real-time imaging make this drug delivery system promising for programmed multimode cancer theranostics.

## Figures and Tables

**Table 1:** Characteristics of DOX-DiR@cerasome (ABC), DOX-DiR@cerasome **(**CA) and DOX-DiR@liposome (ABC), the data were presented as means ± SD (n = 3)

	Diameter (nm)	Polydispersity	Zeta-potential (mV)	Encapsulation efficiency (%)	DOX Loading content (%)	DiR Loading content (%)
DOX-DiR@cerasome (ABC)	45.9±4.3	0.228±0.015	-41.87±0.24	97.70±1.35%	9.40±0.12%	16.03±0.03%
DOX-DiR@cerasome (CA)	42.7±5.3	0.194±0.012	-42.38±0.03	96.58±0.96%	9.64±0.09%	16.05±0.02%
DOX-DiR@lipsome (ABC)	147.8±7.9	0.325±0.009	-41.94±0.1	93.19±4.62%	9.34±0.42%	19.46±0.12%

## References

[1] Torchilin VP (2005). Recent advances with liposomes as pharmaceutical carriers. Nat Rev Drug Discov.

[2] Dromi S, Frenkel V, Luk A, Traughber B, Angstadt M, Bur M (2007). Pulsed-high intensity focused ultrasound and low temperature-sensitive liposomes for enhanced targeted drug delivery and antitumor effect. Clin Cancer Res.

[3] Ponce AM, Vujaskovic Z, Yuan F, Needham D, Dewhirst MW (2006). Hyperthermia mediated liposomal drug delivery. Int J Hyperthermia.

[4] Koning GA, Eggermont AM, Lindner LH, ten Hagen TL (2010). Hyperthermia and thermosensitive liposomes for improved delivery of chemotherapeutic drugs to solid tumors. Pharm Res.

[5] Papahadjopoulos D, Allen TM, Gabizon A, Mayhew E, Matthay K, Huang SK (1991). Sterically stabilized liposomes: improvements in pharmacokinetics and antitumor therapeutic efficacy. Proc Natl Acad Sci U S A.

[6] Dawidczyk CM, Kim C, Park JH, Russell LM, Lee KH, Pomper MG (2014). State-of-the-art in design rules for drug delivery platforms: lessons learned from FDA-approved nanomedicines. J Control Release.

[7] Davis ME, Chen ZG, Shin DM (2008). Nanoparticle therapeutics: an emerging treatment modality for cancer. Nat Rev Drug Discov.

[8] Allen TM, Cullis PR (2004). Drug delivery systems: entering the mainstream. Science.

[9] Kong G, Anyarambhatla G, Petros WP, Braun RD, Colvin OM, Needham D (2000). Efficacy of liposomes and hyperthermia in a human tumor xenograft model: importance of triggered drug release. Cancer Res.

[10] Chang HI, Yeh MK (2012). Clinical development of liposome-based drugs: formulation, characterization, and therapeutic efficacy. Int J Nanomedicine.

[11] Shenoi MM, Shah NB, Griffin RJ, Vercellotti GM, Bischof JC (2011). Nanoparticle preconditioning for enhanced thermal therapies in cancer. Nanomedicine (Lond).

[12] Tagami T, Foltz WD, Ernsting MJ, Lee CM, Tannock IF, May JP (2011). MRI monitoring of intratumoral drug delivery and prediction of the therapeutic effect with a multifunctional thermosensitive liposome. Biomaterials.

[13] Tagami T, Ernsting MJ, Li SD (2011). Optimization of a novel and improved thermosensitive liposome formulated with DPPC and a Brij surfactant using a robust in vitro system. J Control Release.

[14] Chiu GNC, Abraham SA, Ickenstein LM, Ng R, Karlsson G, Edwards K (2005). Encapsulation of doxorubicin into thermosensitive liposomes via complexation with the transition metal manganese. J Control Release.

[15] Banno B, Ickenstein LM, Chiu GNC, Bally MB, Thewalt J, Brief E (2010). The Functional Roles of Poly(Ethylene Glycol)-Lipid and Lysolipid in the Drug Retention and Release from Lysolipid-Containing Thermosensitive Liposomes In Vitro and In Vivo. J Pharm Sci-Us.

[16] Chen KJ, Liang HF, Chen HL, Wang Y, Cheng PY, Liu HL (2013). A thermoresponsive bubble-generating liposomal system for triggering localized extracellular drug delivery. ACS Nano.

[17] Chen KJ, Chaung EY, Wey SP, Lin KJ, Cheng F, Lin CC (2014). Hyperthermia-mediated local drug delivery by a bubble-generating liposomal system for tumor-specific chemotherapy. ACS Nano.

[18] Kang K, Ma J, Yi Q, Gu Z (2017). Localized drug release and effective chemotherapy by hyperthermia-governed bubble-generating hybrid nanocapsule system. Nanomedicine (Lond).

[19] Poon RT, Borys N (2009). Lyso-thermosensitive liposomal doxorubicin: a novel approach to enhance efficacy of thermal ablation of liver cancer. Expert Opin Pharmacother.

[20] Gasselhuber A, Dreher MR, Negussie A, Wood BJ, Rattay F, Haemmerich D (2010). Mathematical spatio-temporal model of drug delivery from low temperature sensitive liposomes during radiofrequency tumour ablation. Int J Hyperthermia.

[21] Negussie AH, Yarmolenko PS, Partanen A, Ranjan A, Jacobs G, Woods D (2011). Formulation and characterisation of magnetic resonance imageable thermally sensitive liposomes for use with magnetic resonance-guided high intensity focused ultrasound. Int J Hyperther.

[22] Yue X, Dai Z (2014). Recent advances in liposomal nanohybrid cerasomes as promising drug nanocarriers. Adv Colloid Interface Sci.

[23] Cao Z, Ma Y, Yue X, Li S, Dai Z, Kikuchi J (2010). Stabilized liposomal nanohybrid cerasomes for drug delivery applications. Chem Commun (Camb).

[24] Kawataki T, Yasuhara K, Kikuchi J (2011). Remarkable Long-term Stability of Cerasome as an Organic-Inorganic Hybrid Nanocontainer for Water-soluble Macromolecules. Chem Lett.

[25] Ma Y, Dai ZF, Gao YG, Cao Z, Zha ZB, Yue XL (2011). Liposomal architecture boosts biocompatibility of nanohybrid cerasomes. Nanotoxicology.

[26] Jin Y, Yue X, Zhang Q, Wu X, Cao Z, Dai Z (2012). Cerasomal doxorubicin with long-term storage stability and controllable sustained release. Acta Biomater.

[27] Cao Z, Yue X, Jin Y, Wu X, Dai Z (2012). Modulation of release of paclitaxel from composite cerasomes. Colloids Surf B Biointerfaces.

[28] Liang X, Li X, Jing L, Xue P, Jiang L, Ren Q (2013). Design and synthesis of lipidic organoalkoxysilanes for the self-assembly of liposomal nanohybrid cerasomes with controlled drug release properties. Chemistry.

[29] Liang X, Gao J, Jiang L, Luo J, Jing L, Li X (2015). Nanohybrid liposomal cerasomes with good physiological stability and rapid temperature responsiveness for high intensity focused ultrasound triggered local chemotherapy of cancer. ACS Nano.

[30] Ke CJ, Su TY, Chen HL, Liu HL, Chiang WL, Chu PC (2011). Smart multifunctional hollow microspheres for the quick release of drugs in intracellular lysosomal compartments. Angew Chem Int Ed Engl.

[31] Ma J, Kang K, Zhang Y, Yi Q, Gu Z (2018). Detachable Polyzwitterion-Coated Ternary Nanoparticles Based on Peptide Dendritic Carbon Dots for Efficient Drug Delivery in Cancer Therapy. ACS Appl Mater Interfaces.

[32] Liu Y, Zhi X, Yang M, Zhang J, Lin L, Zhao X (2017). Tumor-triggered drug release from calcium carbonate-encapsulated gold nanostars for near-infrared photodynamic/photothermal combination antitumor therapy. Theranostics.

[33] Yang Z, Cheng R, Zhao C, Sun N, Luo H, Chen Y (2018). Thermo- and pH-dual responsive polymeric micelles with upper critical solution temperature behavior for photoacoustic imaging-guided synergistic chemo-photothermal therapy against subcutaneous and metastatic breast tumors. Theranostics.

[34] Huang W, Zhao H, Wan J, Zhou Y, Xu Q, Zhao Y (2019). pH- and photothermal-driven multistage delivery nanoplatform for overcoming cancer drug resistance. Theranostics.

[35] Shi S, Liu Y, Chen Y, Zhang Z, Ding Y, Wu Z (2016). Versatile pH-response Micelles with High Cell-Penetrating Helical Diblock Copolymers for Photoacoustic Imaging Guided Synergistic Chemo-Photothermal Therapy. Theranostics.

[36] Li W, Hou W, Guo X, Luo L, Li Q, Zhu C (2018). Temperature-controlled, phase-transition ultrasound imaging-guided photothermal-chemotherapy triggered by NIR light. Theranostics.

[37] Renoux B, Raes F, Legigan T, Peraudeau E, Eddhif B, Poinot P (2017). Targeting the tumour microenvironment with an enzyme-responsive drug delivery system for the efficient therapy of breast and pancreatic cancers. Chem Sci.

[38] Tan Y, Zhu Y, Wen L, Yang X, Liu X, Meng T (2019). Mitochondria-Responsive Drug Release along with Heat Shock Mediated by Multifunctional Glycolipid Micelles for Precise Cancer Chemo-Phototherapy. Theranostics.

[39] Luo Y, Wu H, Feng C, Xiao K, Yang X, Liu Q (2017). "One-Pot" Fabrication of Highly Versatile and Biocompatible Poly(vinyl alcohol)-porphyrin-based Nanotheranostics. Theranostics.

[40] Zhang D, Zheng A, Li J, Wu M, Wu L, Wei Z (2017). Smart Cu(II)-aptamer complexes based gold nanoplatform for tumor micro-environment triggered programmable intracellular prodrug release, photodynamic treatment and aggregation induced photothermal therapy of hepatocellular carcinoma. Theranostics.

[41] Cherukula K, Uthaman S, Park IK (2019). "Navigate-dock-activate" anti-tumor strategy: Tumor micromilieu charge-switchable, hierarchically activated nanoplatform with ultrarapid tumor-tropic accumulation for trackable photothermal/chemotherapy. Theranostics.

[42] O'Neal DP, Hirsch LR, Halas NJ, Payne JD, West JL (2004). Photo-thermal tumor ablation in mice using near infrared-absorbing nanoparticles. Cancer Lett.

[43] Yuan H, Fales AM, Vo-Dinh T (2012). TAT peptide-functionalized gold nanostars: enhanced intracellular delivery and efficient NIR photothermal therapy using ultralow irradiance. J Am Chem Soc.

[44] Moon HK, Lee SH, Choi HC (2009). In vivo near-infrared mediated tumor destruction by photothermal effect of carbon nanotubes. ACS Nano.

[45] Robinson JT, Tabakman SM, Liang YY, Wang HL, Casalongue HS, Vinh D (2011). Ultrasmall Reduced Graphene Oxide with High Near-Infrared Absorbance for Photothermal Therapy. J Am Chem Soc.

[46] Yan F, Duan W, Li Y, Wu H, Zhou Y, Pan M (2016). NIR-Laser-Controlled Drug Release from DOX/IR-780-Loaded Temperature-Sensitive-Liposomes for Chemo-Photothermal Synergistic Tumor Therapy. Theranostics.

[47] Zhou L, Jing Y, Liu Y, Liu Z, Gao D, Chen H (2018). Mesoporous Carbon Nanospheres as a Multifunctional Carrier for Cancer Theranostics. Theranostics.

[48] Jing L, Shao S, Wang Y, Yang Y, Yue X, Dai Z (2016). Hyaluronic Acid Modified Hollow Prussian Blue Nanoparticles Loading 10-hydroxycamptothecin for Targeting Thermochemotherapy of Cancer. Theranostics.

[49] Xiao Z, Ji C, Shi J, Pridgen EM, Frieder J, Wu J (2012). DNA self-assembly of targeted near-infrared-responsive gold nanoparticles for cancer thermo-chemotherapy. Angew Chem Int Ed Engl.

[50] Zhang Z, Wang J, Nie X, Wen T, Ji Y, Wu X (2014). Near infrared laser-induced targeted cancer therapy using thermoresponsive polymer encapsulated gold nanorods. J Am Chem Soc.

[51] Lei Q, Qiu WX, Hu JJ, Cao PX, Zhu CH, Cheng H (2016). Multifunctional Mesoporous Silica Nanoparticles with Thermal-Responsive Gatekeeper for NIR Light-Triggered Chemo/Photothermal-Therapy. Small.

[52] Li C, Yang XQ, Zhang MZ, Song YY, Cheng K, An J (2018). Imaging-Guided Nanoplatform for Tumor Targeting Delivery and Combined Chemo-, Gene- and Photothermal Therapy. Theranostics.

[53] Sun M, Guo J, Hao H, Tong T, Wang K, Gao W (2018). Tumour-homing chimeric polypeptide-conjugated polypyrrole nanoparticles for imaging-guided synergistic photothermal and chemical therapy of cancer. Theranostics.

[54] Zhang J, Zhang J, Li W, Chen R, Zhang Z, Zhang W (2017). Degradable Hollow Mesoporous Silicon/Carbon Nanoparticles for Photoacoustic Imaging-Guided Highly Effective Chemo-Thermal Tumor Therapy and. Theranostics.

[55] Chen H, Liu Z, Li S, Su C, Qiu X, Zhong H (2016). Fabrication of Graphene and AuNP Core Polyaniline Shell Nanocomposites as Multifunctional Theranostic Platforms for SERS Real-time Monitoring and Chemo-photothermal Therapy. Theranostics.

[56] Xia Y, Li W, Cobley CM, Chen J, Xia X, Zhang Q (2011). Gold nanocages: from synthesis to theranostic applications. Acc Chem Res.

[57] Huang XH, El-Sayed IH, Qian W, El-Sayed MA (2006). Cancer cell imaging and photothermal therapy in the near-infrared region by using gold nanorods. J Am Chem Soc.

[58] Yang K, Zhang S, Zhang G, Sun X, Lee ST, Liu Z (2010). Graphene in mice: ultrahigh in vivo tumor uptake and efficient photothermal therapy. Nano Lett.

[59] Xu Q, Wan J, Bie N, Song X, Yang X, Yong T (2018). A Biomimetic Gold Nanocages-Based Nanoplatform for Efficient Tumor Ablation and Reduced Inflammation. Theranostics.

[60] Liao J, Li W, Peng J, Yang Q, Li H, Wei Y (2015). Combined cancer photothermal-chemotherapy based on doxorubicin/gold nanorod-loaded polymersomes. Theranostics.

[61] Li S, Liu Y, Rui Y, Tang L, Achilefu S, Gu Y (2018). Dual target gene therapy to EML4-ALK NSCLC by a gold nanoshell-based system. Theranostics.

[62] Zheng T, Wang W, Wu F, Zhang M, Shen J, Sun Y (2019). Zwitterionic Polymer-Gated Au@TiO Core-Shell Nanoparticles for Imaging-Guided Combined Cancer Therapy. Theranostics.

[63] Xiao Y, Hong H, Matson VZ, Javadi A, Xu W, Yang Y (2012). Gold Nanorods Conjugated with Doxorubicin and cRGD for Combined Anticancer Drug Delivery and PET Imaging. Theranostics.

[64] Jing L, Liang X, Li X, Lin L, Yang Y, Yue X (2014). Mn-porphyrin conjugated Au nanoshells encapsulating doxorubicin for potential magnetic resonance imaging and light triggered synergistic therapy of cancer. Theranostics.

[65] Luo S, Zhang E, Su Y, Cheng T, Shi C (2011). A review of NIR dyes in cancer targeting and imaging. Biomaterials.

[66] Liu H, Wu DC (2016). In vivo Near-infrared Fluorescence Tumor Imaging Using DiR-loaded Nanocarriers. Curr Drug Deliv.

[67] Yu L, Dong A, Guo R, Yang M, Deng L, Zhang J (2018). DOX/ICG Coencapsulated Liposome-Coated Thermosensitive Nanogels for NIR-Triggered Simultaneous Drug Release and Photothermal Effect. ACS Biomater Sci Eng.

[68] Chiu HT, Su CK, Sun YC, Chiang CS, Huang YF (2017). Albumin-Gold Nanorod Nanoplatform for Cell-Mediated Tumoritropic Delivery with Homogenous ChemoDrug Distribution and Enhanced Retention Ability. Theranostics.

[69] Zhao F, Zhou J, Su X, Wang Y, Yan X, Jia S (2017). A Smart Responsive Dual Aptamers-Targeted Bubble-Generating Nanosystem for Cancer Triplex Therapy and Ultrasound Imaging.

[70] Bhattarai P, Liang XL, Xu YX, Dai ZF (2017). A Novel Cyanine and Porphyrin Based Theranostic Nanoagent for Near-Infrared Fluorescence Imaging Guided Synergistic Phototherapy. J Biomed Nanotechnol.

[71] Hashizume M, Kawanami S-i, Iwamoto S, Isomoto T, Kikuchi J-i (2003). Stable vesicular nanoparticle 'Cerasome' as an organic-inorganic hybrid formed with organoalkoxysilane lipids having a hydrogen-bonding unit. Thin Solid Films.

[72] Katagiri K, Hashizume M, Ariga K, Terashima T, Kikuchi J (2007). Preparation and characterization of a novel organic-inorganic nanohybrid "cerasome" formed with a liposomal membrane and silicate surface. Chemistry.

[73] Chung MF, Chen KJ, Liang HF, Liao ZX, Chia WT, Xia Y (2012). A liposomal system capable of generating CO2 bubbles to induce transient cavitation, lysosomal rupturing, and cell necrosis. Angew Chem Int Ed Engl.

[74] Dai Y, Su J, Wu K, Ma W, Wang B, Li M (2019). Multifunctional Thermosensitive Liposomes Based on Natural Phase-Change Material: Near-Infrared Light-Triggered Drug Release and Multimodal Imaging-Guided Cancer Combination Therapy. ACS Appl Mater Interfaces.

[75] Park H, Yang J, Lee J, Haam S, Choi IH, Yoo KH (2009). Multifunctional Nanoparticles for Combined Doxorubicin and Photothermal Treatments. Acs Nano.

[76] Zhang W, Guo ZY, Huang DQ, Liu ZM, Guo X, Zhong HQ (2011). Synergistic effect of chemo-photothermal therapy using PEGylated graphene oxide. Biomaterials.

[77] Liang X, Li X, Jing L, Yue X, Dai Z (2014). Theranostic porphyrin dyad nanoparticles for magnetic resonance imaging guided photodynamic therapy. Biomaterials.

[78] Jing L, Liang X, Li X, Lin L, Yang Y, Yue X (2014). Mn-porphyrin Conjugated Au Nanoshells Encapsulating Doxorubicin for Potential Magnetic Resonance Imaging and Light Triggered Synergistic Therapy of Cancer. Theranostics.

[79] Liang X, Li X, Yue X, Dai Z (2011). Conjugation of Porphyrin to Nanohybrid Cerasomes for Photodynamic Diagnosis and Therapy of Cancer. Angew Chem Int Ed Engl.

[80] You Y, Liang X, Yin T, Chen M, Qiu C, Gao C (2018). Porphyrin-grafted Lipid Microbubbles for the Enhanced Efficacy of Photodynamic Therapy in Prostate Cancer through Ultrasound-controlled In Situ Accumulation. Theranostics.

[81] Su J, Sun H, Meng Q, Yin Q, Zhang P, Zhang Z (2016). Bioinspired Nanoparticles with NIR-Controlled Drug Release for Synergetic Chemophotothermal Therapy of Metastatic Breast Cancer. Adv Funct Mater.

[82] Lin YJ, Huang CC, Wan WL, Chiang CH, Chang Y, Sung HW (2017). Recent advances in CO2 bubble-generating carrier systems for localized controlled release. Biomaterials.

[83] Idris NM, Gnanasammandhan MK, Zhang J, Ho PC, Mahendran R, Zhang Y (2012). In vivo photodynamic therapy using upconversion nanoparticles as remote-controlled nanotransducers. Nat Med.

