# Supplementary material for: Bubble-Manipulated Local Drug Release from a Smart Thermosensitive Cerasome for Dual-Mode Imaging Guided Tumor Chemo-Photothermal Therapy
Source: Theranostics. 2019 Oct 18;9(26):8138–54. doi: 10.7150/thno.36762 (PMC6857040; doi:10.7150/thno.36762)
Supplement: Supplementary file 1 — Supplementary figures and tables. [file thnov09p8138s1.pdf]

# Supporting Information

## Bubble-Manipulated Local Drug Release from a Smart Thermosensitive Cerasome for Dual-Mode Imaging Guided Tumor Chemo-Photothermal Therapy

*Suhui Sun*[#a], *Sujuan Sun*[#b], *Yan Sun*[a], *Ping Wang*[a], *Jianlun Zhang*[a], *Wenjing Du*[a], *Shumin Wang*[*a,b], *Xiaolong Liang*[*a]

[a]Department of Ultrasound, Peking University Third Hospital, Beijing, China

[b]Ordos Center Hospital, Ordos 017000, Inner Mongolia, China

#These authors contributed equally to this work

*Corresponding author

E-mail: xiaolong_liang@bjmu.edu.cn, shuminwang2014@163.com

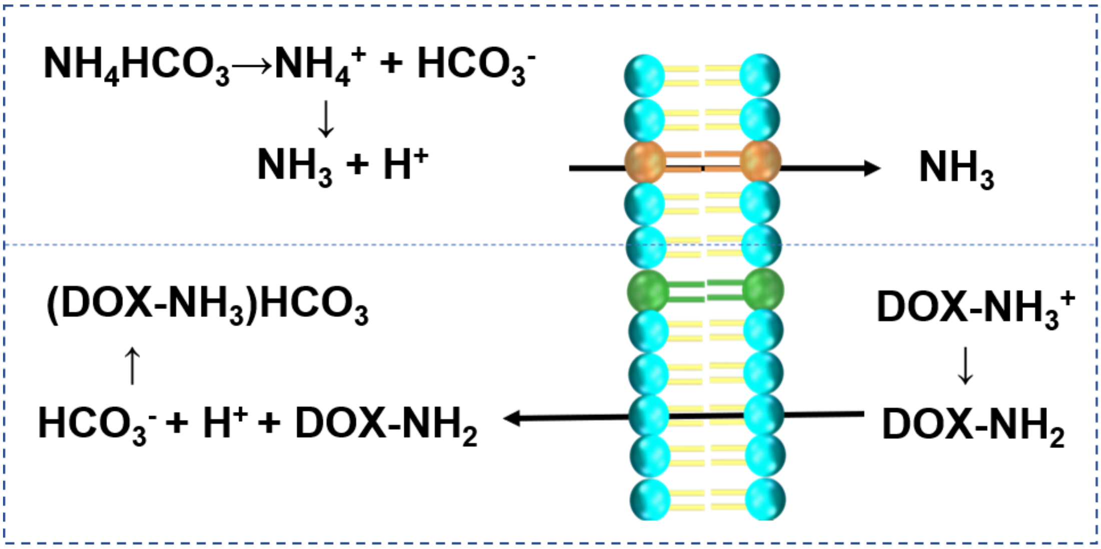


**Figure S1.** Schematic illustrations showing the process of remote loading of DOX.

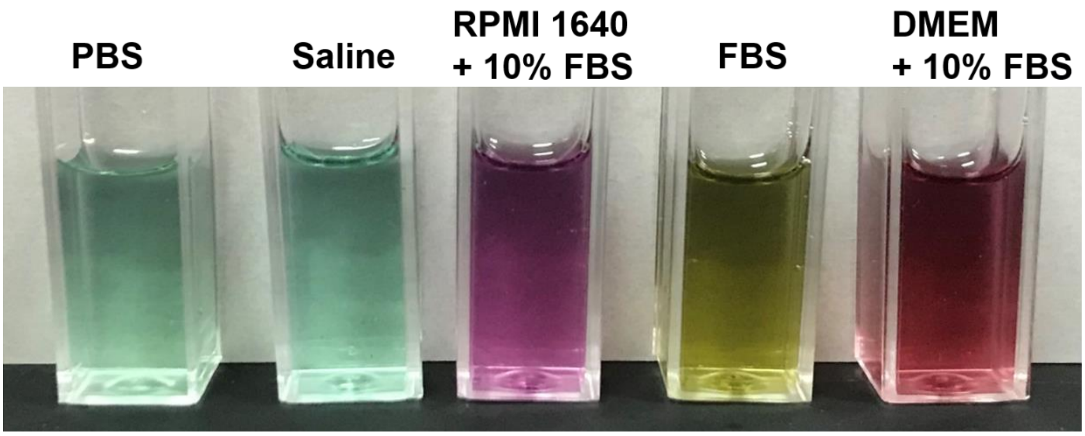


**Figure S2.** Photos of DOX-DiR@cerasome (ABC) dispersed in PBS, Saline and RPMI-1640 culture media containing 10% FBS, fetal bovine serum (FBS) and DMEM culture media containing 10% FBS.

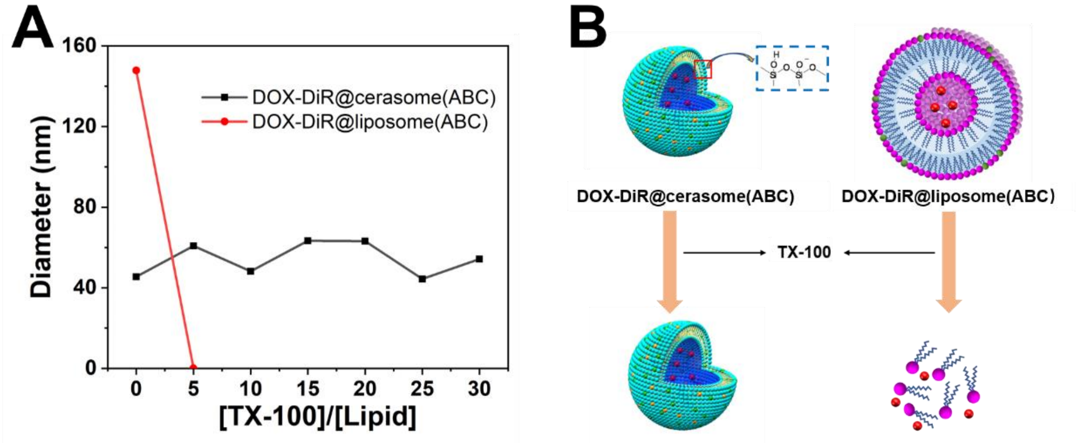


**Figure S3.** Effect of addition of TX-100 on the diameter of DOX-DiR@cerasome (ABC) and DOX-DiR@liposome (ABC).

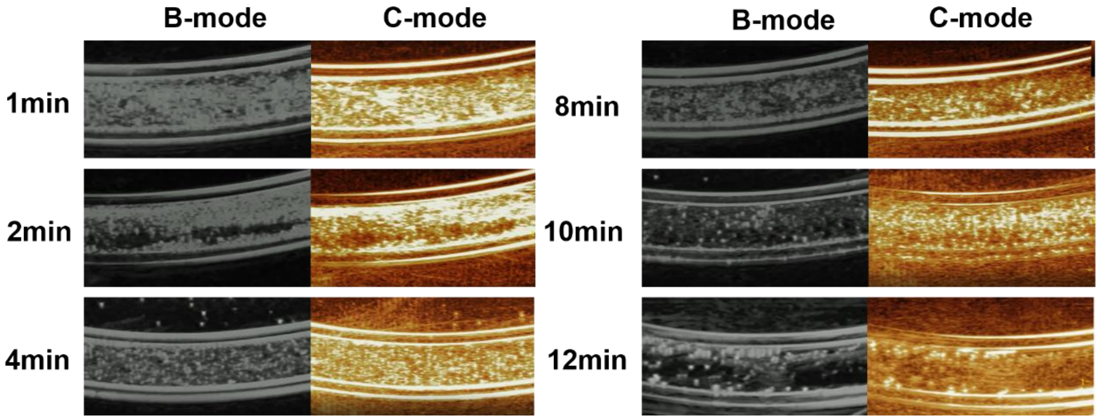


**Figure S4.** *In vitro* US imaging of DOX-DiR@cerasome (ABC) in a test tube at 50℃.

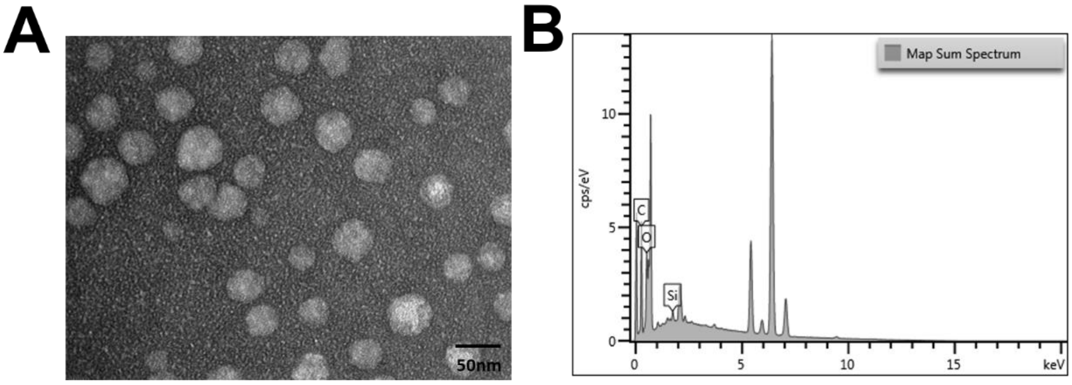


**Figure S5. (A)** TEM micrograph of DOX-DiR@cerasome (ABC) after laser irradiation; **(B)** **S**EM-EDS spectrum of DOX-DiR@cerasome (ABC). scale bar = 50nm.

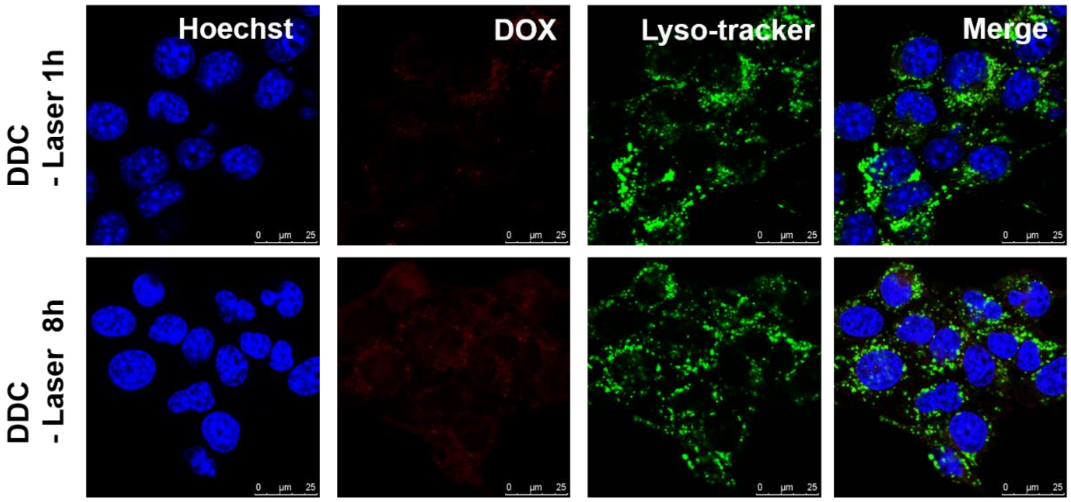


**Figure S6.** Representative CLSM fluorescence images of 4T1 tumor cells after incubation with DOX-DiR@cerasome (ABC) for 4h without laser irradiation, followed by incubation for another 1 h and 8 h, scale bar = 25 μm. (DDC: DOX-DiR@cerasome (ABC)).

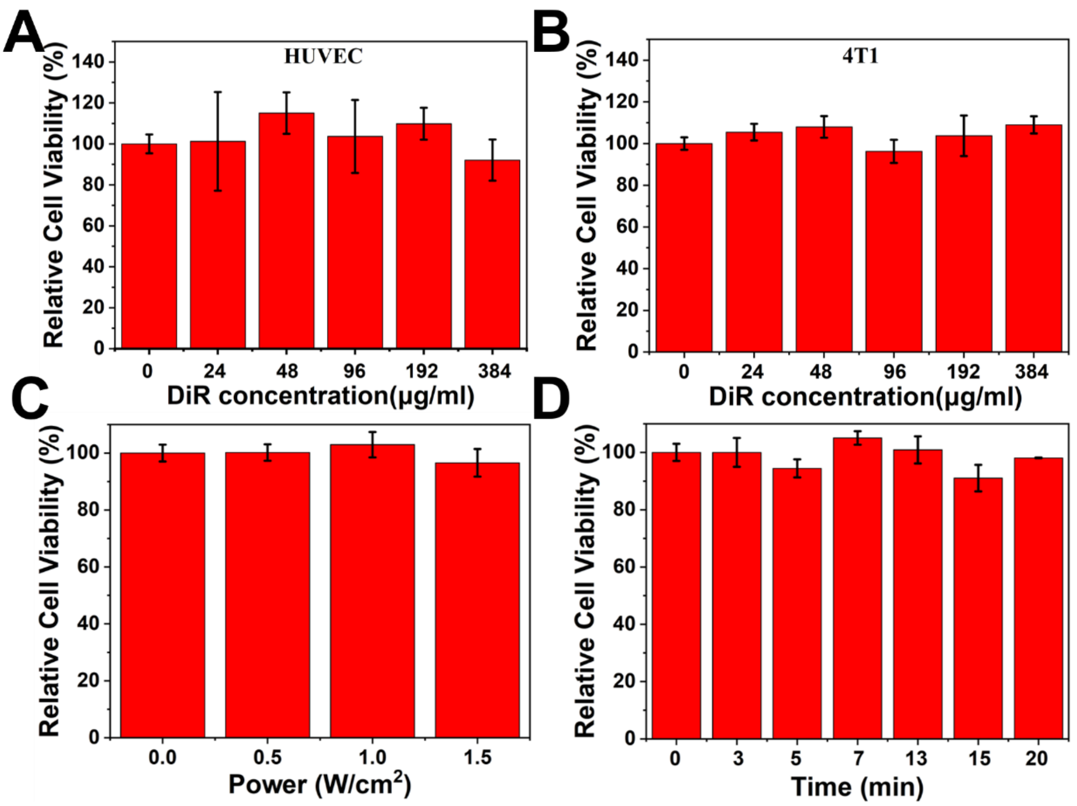


**Figure S7.** Biocompatibility of DiR@cerasome and laser. The cell survival rate of Huvec cells **(A)** and 4T1 cells **(B)** after treatment with different concentration of DiR@cerasome for 72 h as determined by MTT assay. **(C&D)** Cell viability of 4T1 cells after treatment with different power of laser radiation and different time.

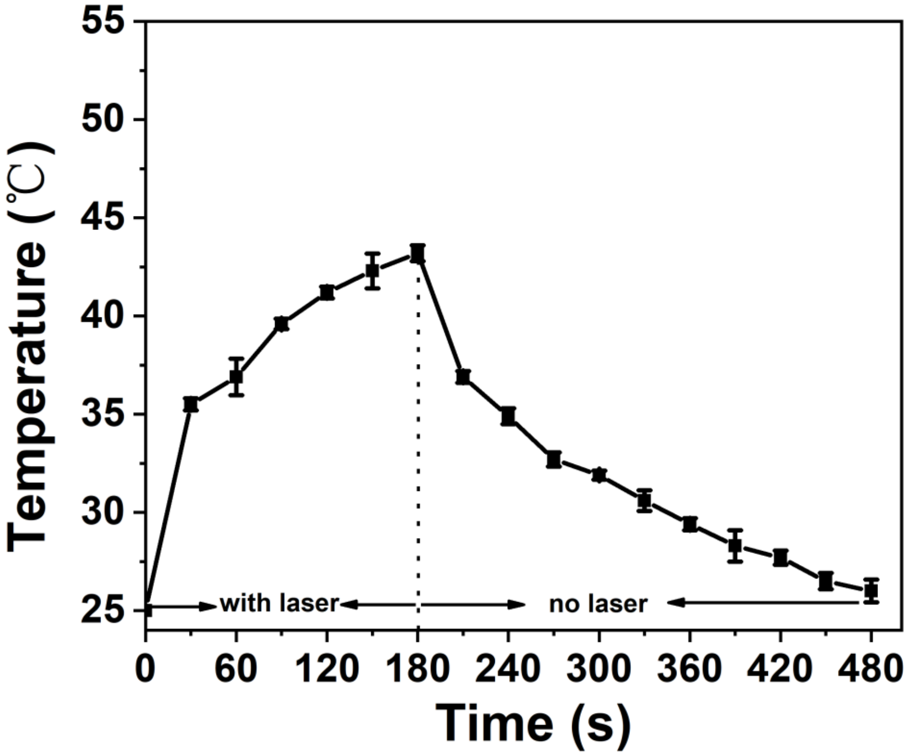


**Figure S8:** Photothermal elevation curve of cells incubated with DiR@cerasome (ABC) at 40μg/ml when irradiated with 760 nm laser. Data shown as means ± SD (n = 3).

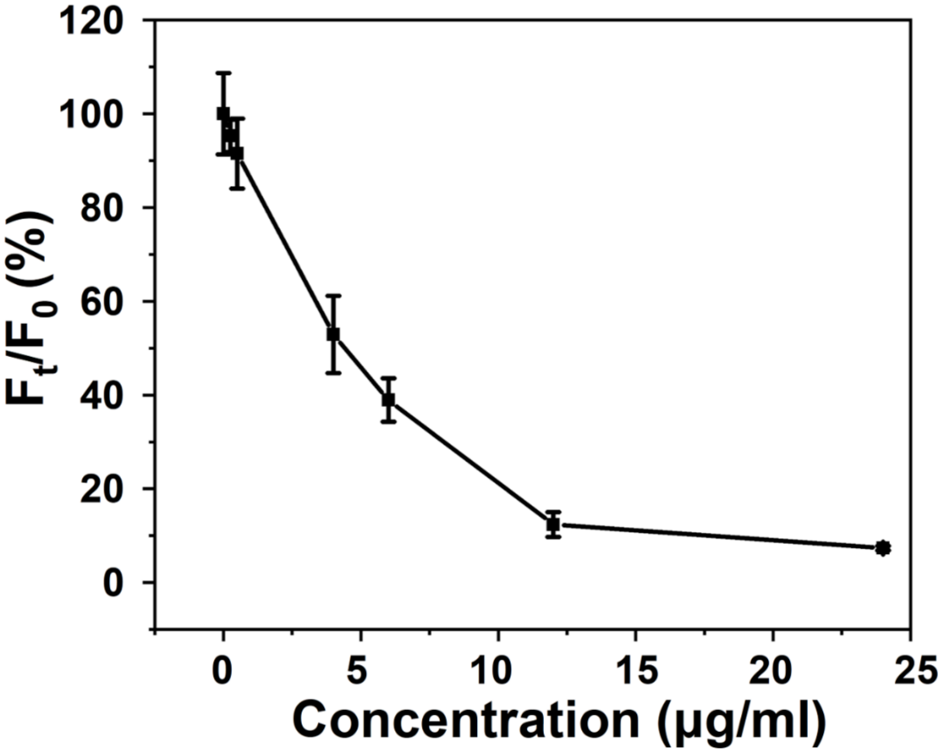


**Figure S9.** Plasma clearance of PGL labelled DOX-DiR@cerasome (ABC) by measuring changes of blood PGL fluorescence (n = 3). $F_0$ and $F_t$ represent the PGL fluorescence intensities at the initial and the given time, respectively.

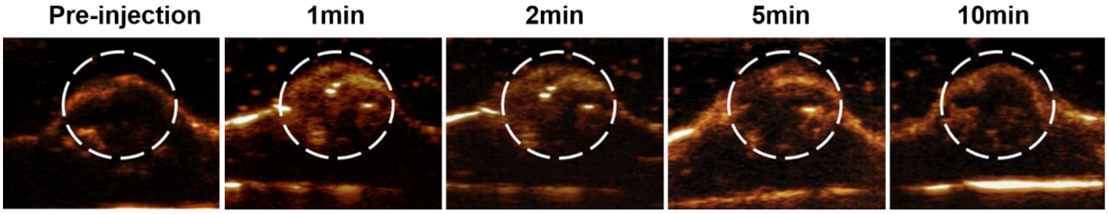


**Figure S10.** Ultrasound imaging of tumor at different times after DOX-DiR@cerasome (ABC)+laser treatment.

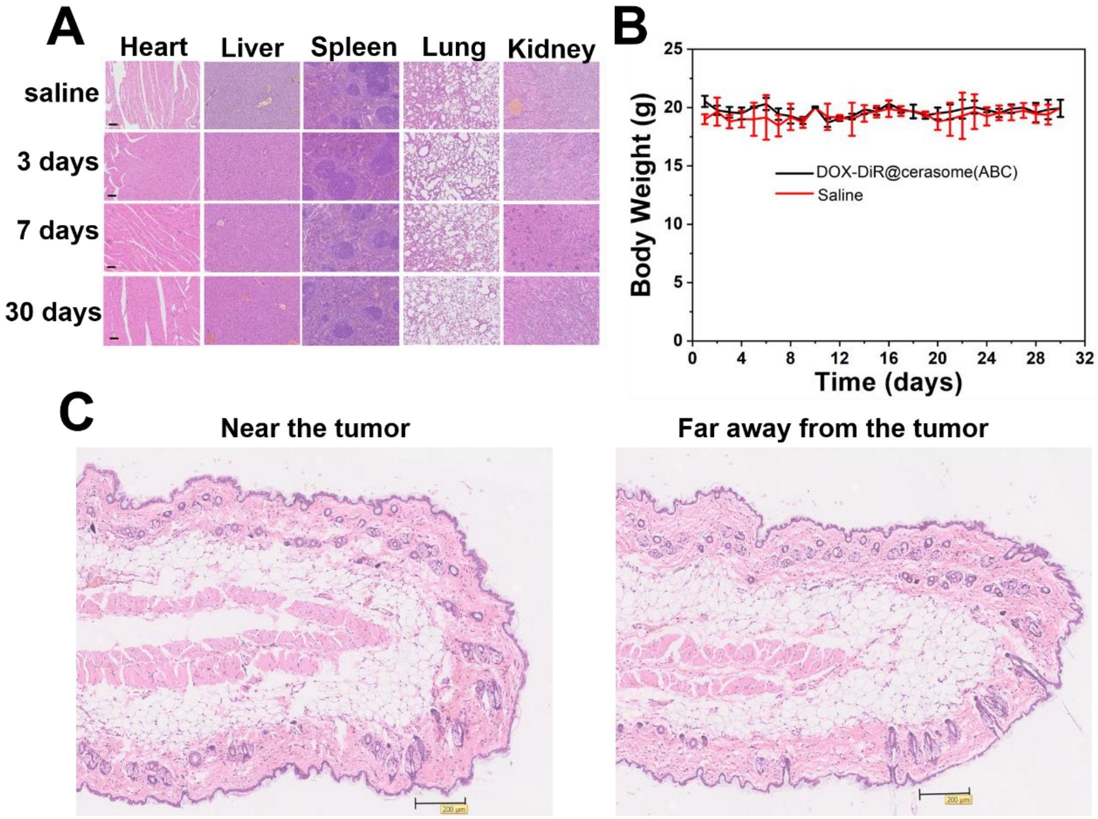


**Figure S11. A)** Histological analysis of vital organs (heart, liver, spleen, lung and kidney) with H&E staining at 3, 7 and 30 days after intravenous administration DOX-DiR@cerasome (ABC) into the healthy Balb/c mice (DOX concentration at 5 mg/kg). Mice treated with saline were used as control. Scale bar = 100 μm. **B)** Changes of body weight with increasing time. **C)** Histological analysis of tissues near and far away from tumor with H&E staining different treatments. Scale bar = 200 μm.
